# Engineering extracellular vesicles by three‐dimensional dynamic culture of human mesenchymal stem cells

**DOI:** 10.1002/jev2.12235

**Published:** 2022-06-18

**Authors:** Xuegang Yuan, Li Sun, Richard Jeske, Dingani Nkosi, Sara B. York, Yuan Liu, Samuel C. Grant, David G. Meckes, Yan Li

**Affiliations:** ^1^ Department of Chemical and Biomedical Engineering Florida State University Tallahassee Florida USA; ^2^ Present address: Broad Stem Cell Research Center, David Geffen School of Medicine University of California‐Los Angeles (UCLA) Los Angeles CA USA; ^3^ The National High Magnetic Field Laboratory Tallahassee Florida USA; ^4^ Department of Biomedical Sciences College of Medicine Tallahassee Florida USA

**Keywords:** human mesenchymal stem cells, extracellular vesicles, exosome, 3D aggregation, rejuvenation, immunomodulation, biomanufacturing

## Abstract

Human mesenchymal stem cell (hMSC) derived extracellular vesicles (EVs) have shown therapeutic potential in recent studies. However, the corresponding therapeutic components are largely unknown, and scale‐up production of hMSC EVs is a major challenge for translational applications. In the current study, hMSCs were grown as 3D aggregates under wave motion to promote EV secretion. Results demonstrate that 3D hMSC aggregates promote activation of the endosomal sorting complexes required for transport (ESCRT)‐dependent and ‐independent pathways. mRNA sequencing revealed global transcriptome alterations for 3D hMSC aggregates. Compared to 2D‐hMSC‐EVs, the quantity of 3D‐hMSC‐EVs was enhanced significantly (by 2‐fold), with smaller sizes, higher miR‐21 and miR‐22 expression, and an altered protein cargo (e.g., upregulation of cytokines and anti‐inflammatory factors) uncovered by proteomics analysis, possibly due to altered EV biogenesis. Functionally, 3D‐hMSC‐EVs rejuvenated senescent stem cells and exhibited enhanced immunomodulatory potentials. In summary, this study provides a promising strategy for scalable production of high‐quality EVs from hMSCs with enhanced therapeutic potential.

## 1 INTRODUCTION

Human mesenchymal stem/stromal cells (hMSCs) derived from various tissue sources are widely acknowledged as a promising candidate for stem‐cell therapy in stroke, Alzheimer's disease multiple sclerosis, myocardiac disease, graft‐versus‐host disease, infectious diseases and others (Yin et al., 2019). Despite their multilineage differentiation potential, hMSCs exert therapeutic effects via their secretome and paracrine factors (Yin et al., 2019). Within the MSC secretome, extracellular vesicles (EVs) are of great interest, as recent results have shown their therapeutic potential in multiple disease models (Wiklander et al., 2019). EVs are defined as small (a size of 20–500 nm), membrane‐enclosed particles released from cells that play an essential role in intercellular communication due to their capacity to transfer membrane constituents and cargoes of cytosolic proteins, lipids, metabolites and genetic materials (e.g., microRNAs) (Deng et al., 2018; Jeppesen et al., 2019; Liu et al., 2018; van Niel et al., 2018; Zhang et al., 2019). The internal payload and membrane composition of hMSC EVs may have therapeutic benefits in treating neurodegeneration. (Gyorgy et al., 2015; Jarmalavičiūtė & Pivoriūnas, 2016; Osorio‐Querejeta et al., 2018; Riazifar et al., 2017; Wiklander et al., 2019). hMSC EVs also have been reported to promote tissue regeneration in ischemic diseases and exert anti‐aging effects by reducing cellular oxidative stress (Baek et al., 2019; Branscome et al., 2019; Ha et al., 2016; Keshtkar et al., 2018; Riazifar et al., 2017; Zhang et al., 2019). Preclinical studies have shown that EVs derived from hMSCs reduce oxidative stress, ameliorate inflammation, and promote oligodendrocyte formation and re‐myelination in an experimental autoimmune encephalomyelitis (EAE) model of demyelination for multiple sclerosis (Laso‐Garcia, 2018; Osorio‐Querejeta et al., 2018). In preclinical ischemic stroke, hMSC EVs promoted an anti‐inflammatory and anti‐apoptosis response as well as angiogenesis at the lesion site, showing a considerably decreased infarct volume and improved motor functions (Kim et al., 2020; Xin et al., 2013; Zhang et al., 2019). In an Alzheimer's disease model, better cognitive behaviour and lower Aβ plaque load in the hippocampus were observed in hMSC EV‐treated mice (Cone et al., 2021; Elia et al., 2019). While these results demonstrated the potential for hMSC EVs in treating various neurological disorders, the correlations between parental cell transcriptome and hMSC EV cargo profiles of proteins, microRNAs, or metabolites, and neurological regeneration capacity have not been elucidated fully with respect to in vitro culture conditions.

It has been reported that the hMSC EV properties can be mediated by the microenvironment and in vitro conditions, such as hypoxia or hydrodynamic culture in bioreactors (Patel et al., 2017; Patel et al., 2018). In particular, 3D aggregation, as a preconditioning strategy, is expected to promote hMSC EV production with beneficial cargo profile compared to 2D culture. For instance, HSP90 and EpCAM expression were highly promoted in the secreted EVs of 3D cancer organoids compared to 2D generated EVs (Eguchi et al., 2018), and the 3D EVs better reflected the characteristics of in vivo exosomes, a subpopulation of EVs with a size range of 30–200 nm (Villasante et al., 2016). The cargo profiles of EVs from 3D cortical spheroids derived from human induced pluripotent stem cells (hiPSCs) have been reported to reflect lineage‐specific developmental stages of stem cell differentiation in previous studies (Marzano et al., 2019; Marzano et al., 2021). Moreover, recent studies provide strong evidence that 3D aggregate culture induces drastic changes in hMSC homeostasis, characterized by enhanced glycolysis, activation of autophagy, and anti‐senescence property, retention of the primitive phenotype of hMSCs in vitro (Bijonowski et al., 2020; Bijonowski et al., 2020; Liu et al., 2017; Sart et al., 2014; Yuan et al., 2019; Yuan et al., 2019). In particular, the secretome of 3D hMSC aggregates exhibited enhanced expression of cytokines associated with immunosuppression and anti‐apoptosis compared to 2D culture (Bartosh et al., 2010; Sart et al., 2014). While 3D aggregation of hMSCs has attracted growing interest to enhance therapeutic outcomes (Sart et al., 2014), the properties of EVs secreted by 3D hMSC aggregates have not been well investigated. As EVs carry a majority of cellular characteristics of the parental cells, it is hypothesized that EVs from 3D hMSC aggregates may exhibit characteristics for better therapeutic benefits.

In this study, characteristics of hMSC EVs derived from 3D aggregates (3D‐hMSC‐EVs) and 2D planer culture (2D‐hMSC‐EVs) were investigated thoroughly. Bone marrow derived hMSCs were aggregated spontaneously in a miniaturized dynamic wave‐motion bioreactor, as reported in our previous study (Tsai et al., 2017). hMSC‐EVs were isolated and characterized for yield, size distribution, EV markers, protein cargo and miRNA cargo. Moreover, various in vitro functional assays were performed, including the anti‐aging effects of 3D EVs on adult stem cells with replicative senescence, the immunomodulatory effects of 3D EVs on macrophage polarization, and the inhibition of T cell proliferation, as well as an in vitro wound healing model. To reveal the possible relevant pathways, global transcriptome of 2D and 3D hMSCs was examined by mRNA sequencing along with the proteomics analysis of the EV protein cargo. These analyses provide correlations between the parental cell transcriptome with secreted EV protein cargo as well as the functional outcomes of the EV‐recipient cells. Together, these results indicate that 3D aggregation of hMSCs significantly enhances the secretion of EVs compared to 2D adherent culture. The secreted 3D‐hMSC‐EVs may possess enhanced therapeutic potentials compared with 2D EVs, likely due to the cargo differences. The enhanced EV properties with respect to immunomodulation and regulatory homeostasis are beneficial for treating neurological disorders potentially by promoting the secretion of neuroprotective factors and suppressing inflammatory response, in a way similar to brain pericytes (Crisan et al., 2008). The existence of similarities between hMSCs and brain pericytes alludes to the presence of hMSCs in adult brain (Crisan et al., 2008; da Silva Meirelles et al., 2006). Moreover, the scalable bioreactor culture approach enables scale‐up processes for biomanufacturing of hMSC EVs for future clinical applications.

## 2 RESULTS

### 2.1 Global transcriptome analysis of 3D aggregated hMSCs

**FIGURE 1:**
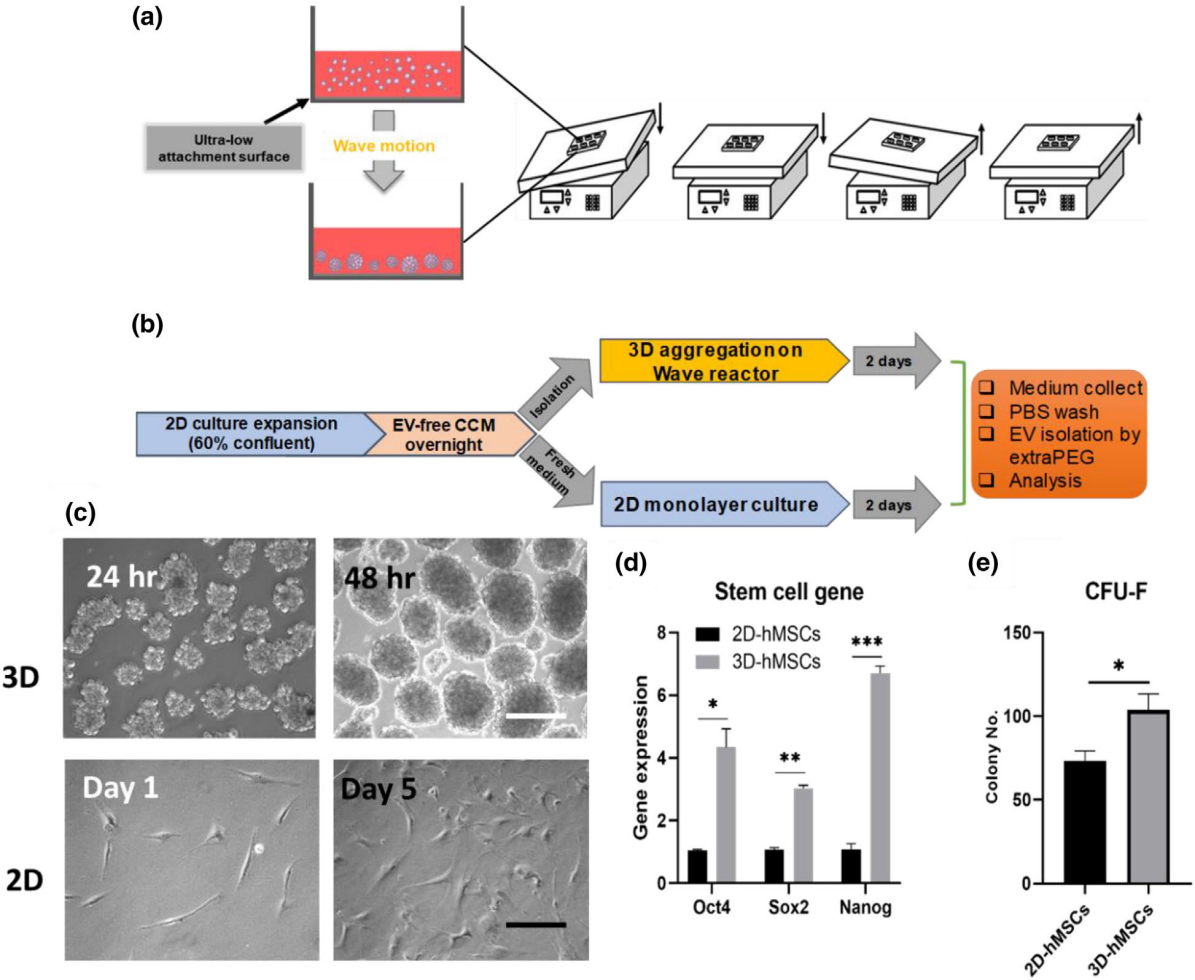
Culture conditions for 2D and 3D hMSCs to promote EV secretion. (a) Illustration of 3D dynamic culture of hMSCs to form cell aggregates using a miniaturized wave bioreactor. (b) Schematic illustration of procedures from initial 2D expansion of hMSCs to the medium collection of 2D and 3D cultures and isolation of 2D‐hMSC‐EVs and 3D‐hMSC‐EVs. (c) Representative images of 3D hMSC aggregates and 2D culture of hMSCs. Scale bar: 200 μm (white), 50 μm (black). (d) Stem cell gene expression and (e) Colony forming unit‐fibroblast (CFU‐F) colony number from 2D or dynamic 3D culture of hMSCs in EV‐depleted complete culture medium (CCM). *n* = 3. *P < 0.05; **P < 0.01; ***P < 0.001

Figure 1 illustrates the miniaturized Wave Bioreactor and experimental procedures of 3D hMSC cultures for the generation of 3D‐hMSC‐EVs in comparison to 2D hMSC cultures (Figure 1a and b). Generally, 3D dynamic culture generated a large amount of hMSC aggregates with relatively narrow size distribution (Figure 1c). The dynamic culture of Wave Motion Bioreactor also promoted the expression of stemness genes *Oct4, Sox2* and *Nanog* in hMSCs as well as increased their colony forming unit‐fibroblasts (CFU‐F) activity, reflecting a more primitive phenotype compared to 2D culture (Figure 1d and e). Although cell‐cell and cell‐matrix interactions exist in 3D aggregation culture, it is the stress response to cellular re‐organization and metabolic reconfiguration that maintain the primitive and quiescent phenotype of hMSCs based on our previous studies (Bijonowski et al., 2020; Liu et al., 2017; Liu et al., 2019). In addition, the 3D aggregated hMSCs demonstrated lineage‐specific differentiation ability (Figure S1).

**FIGURE 2:**
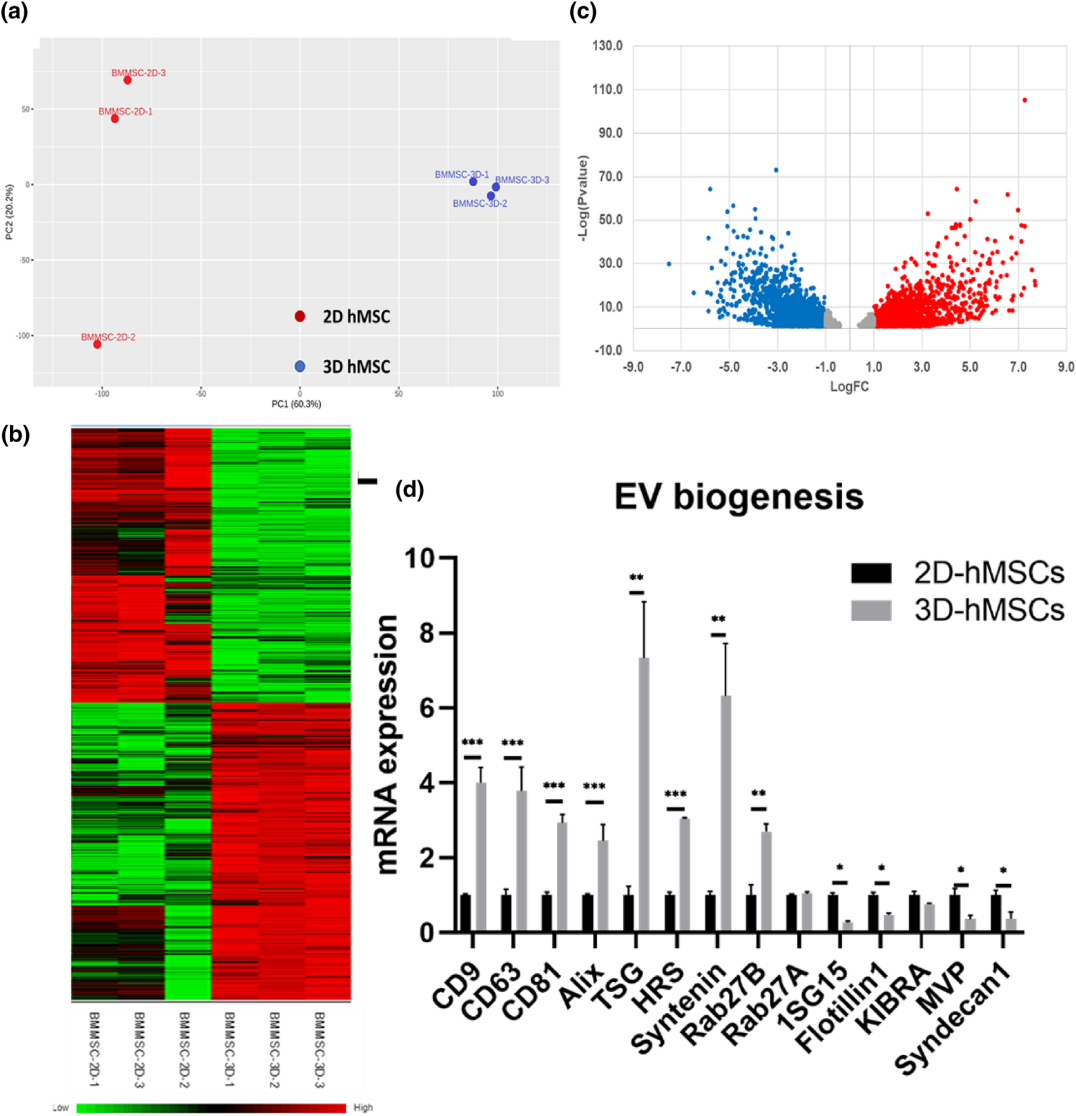
mRNA sequencing of 2D vs. 3D hMSCs and EV biogenesis marker expression. (a) Principal component analysis (PCA) plots of different culture conditions (2D and 3D) in triplicate. (b) Heatmap of differentially expressed genes (DEGs) from mRNA sequencing. (c) Volcano plots illustrate the number of significant DEGs (3D/2D). Red colour symbols indicate the upregulated DEGs and blue symbols indicate the downregulated DEGs. (d) Genes involved in EV biogenesis and secretion pathways in hMSCs determined by qRT‐PCR. *n* = 3. *P < 0.05; **P < 0.01; ***P < 0.001

Transcriptome analysis of 2D and 3D bone marrow hMSCs with triplicate libraries (total 6; Figure 2, data file S1 and S2) as well as umbilical cord (UC) hMSCs from different donors, that is, six libraries for 2D (Donor‐2D) and six for 3D (Donor‐3D) (total 12; Figure S1), was performed using next‐generation sequencing of mRNA. Principal component analysis (PCA) shows a significant separation between 3D and 2D conditions, and the replicates generally exhibit a tight cluster for each group (Figure 2a). Differences between 3D and 2D conditions were not affected by different donors of hMSCs as shown in a separate PCA plot (Figure S2). Compared to 2D conditions, 3D culture induces significant transcriptome alterations for hMSCs from different tissue source and donors. The heatmap of differentially expressed genes (DEGs) also shows significant differences between 3D and 2D hMSCs (Figure 2b). Volcano plots show a total of 3109 DEGs (*P* value < 0.05, Log2 fold change > 1.0) between 3D and 2D conditions (Figure 2c). The upregulated DEGs and the downregulated DEGs were comparable (i.e., DEG distribution and the expression levels) for 3D‐ versus 2D‐hMSCs. The top 20 genes that are upregulated or downregulated DEGs are listed in Table S1. Kyoto Encyclopaedia of Genes and Genomes (KEGG) pathway analysis shows DEG‐enriched pathways by comparing 3D to 2D hMSCs (Table 1). KEGG analysis identified the mitogen‐activated protein kinase (MAPK), Ras, Wnt signalling, cell cycle, and apoptosis pathways, implying that 3D dynamic culture systematically alters the hMSC transcriptome.

**TABLE 1:** The top 20 enriched KEGG pathways from DEGs between 3D and 2D hMSCs based on RNA‐seq

Pathway	Hits	P value	FDR
Pathways in cancer	114	3.32E‐06	0.00105
Focal adhesion	51	1.86E‐05	0.00198
Hippo signalling pathway	42	2.06E‐05	0.00198
Proteoglycans in cancer	51	2.49E‐05	0.00198
MAPK signalling pathway	68	3.71E‐05	0.00208
Pathogenic Escherichia coli infection	20	4.27E‐05	0.00208
Cell cycle	35	4.58E‐05	0.00208
Axon guidance	46	5.90E‐05	0.00218
Cytokine‐cytokine receptor interaction	67	6.16E‐05	0.00218
Basal cell carcinoma	21	0.00012	0.00381
Apoptosis	36	0.000153	0.00444
Breast cancer	38	0.000174	0.00444
Leukocyte transendothelial migration	31	0.000182	0.00444
Ferroptosis	15	0.000259	0.00589
Rap1 signalling pathway	48	0.00039	0.00797
Wnt signalling pathway	39	0.000401	0.00797
Mineral absorption	17	0.000522	0.00977
cGMP‐PKG signalling pathway	40	0.000574	0.0101
Ras signalling pathway	52	0.000611	0.0102
Protein digestion and absorption	25	0.000701	0.0111

Abbreviations: KEGG, Kyoto Encyclopedia of Genes and Genomes; DEG, differentially expressed gene; FDR, permutation‐based false‐discovery rate.

An FDR value is a *P*‐value adjusted for multiple tests (by the Benjamini‐Hochberg procedure).

To understand the global transcriptional change under 3D culture, certain DEGs are identified in the categories of cytoskeleton, extracellular matrix (ECM), cytokine and EV biogenesis (Table 2). Cytoskeleton and cadherin proteins are all downregulated, potentially due to changes in cell morphology, reduced cell‐matrix adhesion and reorganized cytoskeletons (Bijonowski et al., 2019; Tsai et al., 2015). Differential expressions of claudins and other gap junction proteins also are associated with cellular re‐organization under dynamic 3D culture. On the other hand, upregulated matrix metalloproteinases imply extensive remodelling of ECM proteins. The higher levels of *FGF, TGFB, VEGFA* and *IL‐10* chemokines indicate that 3D‐cultured hMSCs may promote the cytokine and chemokine secretion compared to standard 2D cultures. For instance, IL‐10 is a potent anti‐inflammatory and immunosuppression cytokine, which has 3.9‐fold increase in 3D hMSCs. In addition, CXCLs (i.e., CXCL1, 2, 3, 5, 6, 8, 12 and 16) were upregulated in 3D hMSCs (Table S2).

**TABLE 2:** The selected DEGs between 3D and 2D hMSCs from RNA‐seq

Category	Symbols	Name	Log FC	*P* value
Cytoskeleton	TUBA1B	tubulin alpha 1b	−3.24	4.60E‐43
	TUBB2A	tubulin beta 2A class IIa	−2.76	2.77E‐29
	ACTA2	actin alpha 2, smooth muscle	−4.47	4.88E‐27
	ACTC1	actin alpha cardiac muscle 1	−5.93	1.49E‐17
	ACTB	actin beta	−2.41	3.00E‐31
	ACTG1	actin gamma 1	−2.06	2.53E‐16
ECM	CDH1	cadherin 1	−1.33	3.77E‐02
	CDH2	cadherin 2	−1.36	4.98E‐04
	CLDN1	claudin 1	−1.91	7.51E‐03
	CLDN10	claudin 10	3.15	2.36E‐04
	MMP1	matrix metallopeptidase 1	5.62	6.65E‐18
	MMP13	matrix metallopeptidase 13	6.72	2.77E‐33
	MMP3	matrix metallopeptidase 3	5.46	6.02E‐08
	MMP9	matrix metallopeptidase 9	5.92	5.91E‐08
Cytokine	FGF1	fibroblast growth factor 1	1.21	1.36E‐03
	FGF13	fibroblast growth factor 13	2.54	2.78E‐02
	IL10	interleukin 10	3.92	2.38E‐03
	TGFB1	transforming growth factor beta 1	1.64	1.18E‐05
	TGFB2	transforming growth factor beta 2	1.13	8.57E‐04
	VEGFA	vascular endothelial growth factor A	2.51	8.89E‐14
EV biogenesis	RAB27B	RAB27B, member RAS oncogene family	3.37	2.08E‐20
	CD9	CD9 molecule	1.41	6.58E‐03
	SDCBP	syndecan binding protein	0.90	1.62E‐02
	VPS37B	VPS37B subunit of ESCRT‐I	0.75	6.88E‐03
	CHMP4A	charged multivesicular body protein 4A	−0.44	4.28E‐02
	CHMP7	charged multivesicular body protein 7	0.68	6.51E‐05
	CERK	ceramide kinase	1.36	3.14E‐09

*Note*: The numbers are the Log2 values of 3D to 2D hMSCs. Negative values (Green) indicate that the genes are present in higher amounts in the 2D group, while positive values (Red) indicate that the genes are present in higher amounts in the 3D group. DEG: differentially expressed gene.

Understanding hMSC secretome and EV biogenesis mechanisms are of significant interest due to the great potential of hMSC EVs as therapeutic delivery systems. Several key components in endosomal sorting complexes required for transport (ESCRT)‐dependent, ESCRT‐independent and ceramide pathways are upregulated in 3D hMSCs (Table 2). EV biogenesis genes differentially expressed as determined by RNA‐Seq were verified by qRT‐PCR for the 3D and 2D hMSCs (Figure 2d). *CD9, CD63* and *CD81* are upregulated significantly in 3D hMSCs (3‐4 fold), contributing to enhanced ESCRT‐independent pathway cargo sorting and EV biogenesis. *Alix, TSG, HRS*, *Syntenin* and *Rab27B* also are upregulated significantly (2.5‐7 fold), facilitating ESCRT‐dependent pathways. For example, Rab27B is a small GTPase family member that facilitates multi‐vesicular body (MVB) fusion with the plasma membrane and exosomal release. Overexpression of *Rab27* increases EV secretion, and knockdown of *Rab27* reduces EV yield (Ostrowski et al., 2010). Another study has shown that 3D EVs of cancer cells have different miRNA and protein cargo from 2D EVs, but did not analyze the EV biogenesis markers (Rocha et al., 2019). Therefore, dynamic 3D aggregate culture alters the EV biogenesis in hMSCs by potentially activating both ESCRT‐dependent and ‐independent pathways.

The intracellular miRNA profiles were validated by qRT‐PCR on the hMSCs from 2D and 3D cultures (Figure S3). Upregulation was observed for miR‐18a‐5p, miR‐23a‐3p, miR‐29b‐3p, miR‐100‐5p, miR‐125b‐5p, miR‐146a‐5p, miR‐155‐5p, miR‐199a‐3p, miR‐630 and miR‐4454. Down‐regulation was found for miR‐17‐3p, miR‐19a‐3p, miR‐19b‐3p and miR‐328‐3p. These miRNAs regulate various cellular behaviours and signalling pathways (e.g., miR‐155 in activation of Wnt pathway, miR‐29b and miR‐199a in regulating matrix metalloproteinase, and miR‐19a in inhibiting Cyclin D1) (Jeske et al., 2020; Song et al., 2015). Therefore, 3D dynamic aggregate culture altered the intracellular miRNA profile of hMSCs and potentially may impact exosomal cargo packaging. Taken together, the distinct transcriptome characteristics of hMSCs under 3D dynamic culture may influence the EV biogenesis and properties.

### 2.2 3D dynamic culture promotes EV generation from hMSCs

**FIGURE 3:**
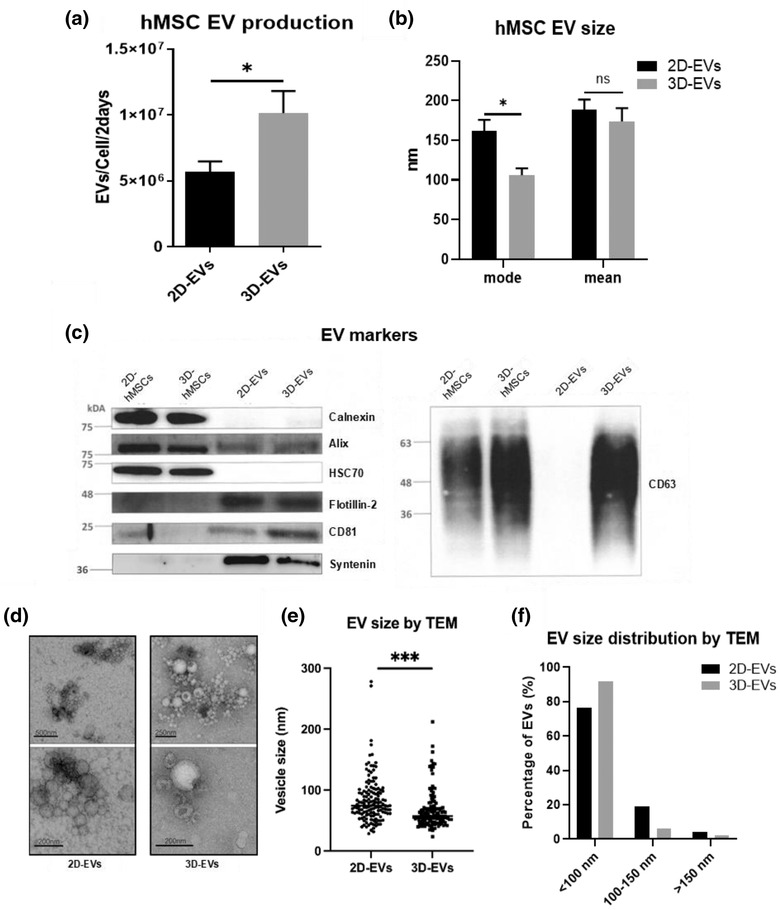
Characterization of hMSC EVs derived from 2D monolayer culture and 3D dynamic culture. (a) EV production from 2D and 3D culture at 2‐day interval determined by nanoparticle tracking analysis (NTA), normalized to cell number at day 2 (*n* = 6). (b) The mean and mode size of EVs (*n* = 6). (c) Positive and negative markers of 2D EVs and 3D EVs as well as corresponding cell lysate determined by Western Blot. (d) Transmission electron microscopy (TEM) images of 2D EVs and 3D EVs. Scale bars are indicated in the images. (e) EV size (*n* = 150) and (f) distribution determined based on TEM images. *P < 0.05; ***P < 0.001. ns: not significant

The secreted 2D‐ and 3D‐hMSC‐EVs were characterized by nanoparticle tracking analysis (NTA), Western blot and transmission electronic microscopy (TEM; Figure 3). From NTA, the 3D dynamic aggregation culture results in two‐fold higher numbers of EVs per cell‐base than the 2D monolayer culture at a 2‐day interval (Figure 3a). While average mean size of EVs are comparable (∼180 nm) for 2D‐ and 3D‐hMSC‐EVs, the average mode size of 3D‐hMSC‐EVs is significantly lower than the 2D‐hMSC‐EVs (100 nm vs. 155 nm) (Figure 3b). The mode size is considered as a more accurate representation because vesicle aggregates may affect the value of the mean size. Exosomal markers were evaluated via Western Blot (Figure 3c), showing the expression of positive EV markers Alix, Flotillin‐2, CD81 (higher in 3D‐hMSC‐EVs) and Syntenin (higher in 2D‐hMSC‐EVs), as well as the absence of Calnexin (a negative EV marker). HSC70 expression is weak for both 2D‐ and 3D‐hMSC‐EVs (Figure S4), which also was observed in previously isolated EVs by the ExtraPEG method (Hurwitz et al., 2018; Rider et al., 2016). In particular, the CD63 (a tetraspanin commonly found on the surface of exosomes) expression is much higher in 3D‐hMSC‐EVs than 2D‐hMSC‐EVs (Figure S4). The TEM images verified the presence of exosome‐sized, cup‐shaped vesicles (Figure 3d). Analysis of TEM images confirmed that the 3D‐hMSC‐EVs are significantly smaller compared to 2D‐hMSC‐EVs (55 nm vs. 75 nm, on average) (Figure 3e and f). These results indicate that dynamic 3D aggregation enhances hMSC EV production and enriches the fractions of small EVs (i.e., exosomes), which have been proposed as the major contributor of the therapeutic potential of hMSC EVs (Murphy et al., 2019).

To confirm the enhanced EV production in dynamic 3D aggregate culture, bone marrow hMSCs from a different donor, at different passages, as well as human adipose‐derived stem cells (ASCs) and UC‐hMSCs were evaluated for 3D EV production (Figures S5 and S6). Consistently for each cell source and tissue origin, significantly higher EV yield (EVs/Cell/2 days) was observed for dynamic 3D aggregate culture compared to the corresponding 2D monolayer culture. The mode size of EVs was also relatively smaller for 3D‐hMSC‐EVs compared to 2D‐hMSC‐EVs.

### 2.3 3D dynamic aggregates promote therapeutically relevant miRNA and protein cargo in EVs

**FIGURE 4:**
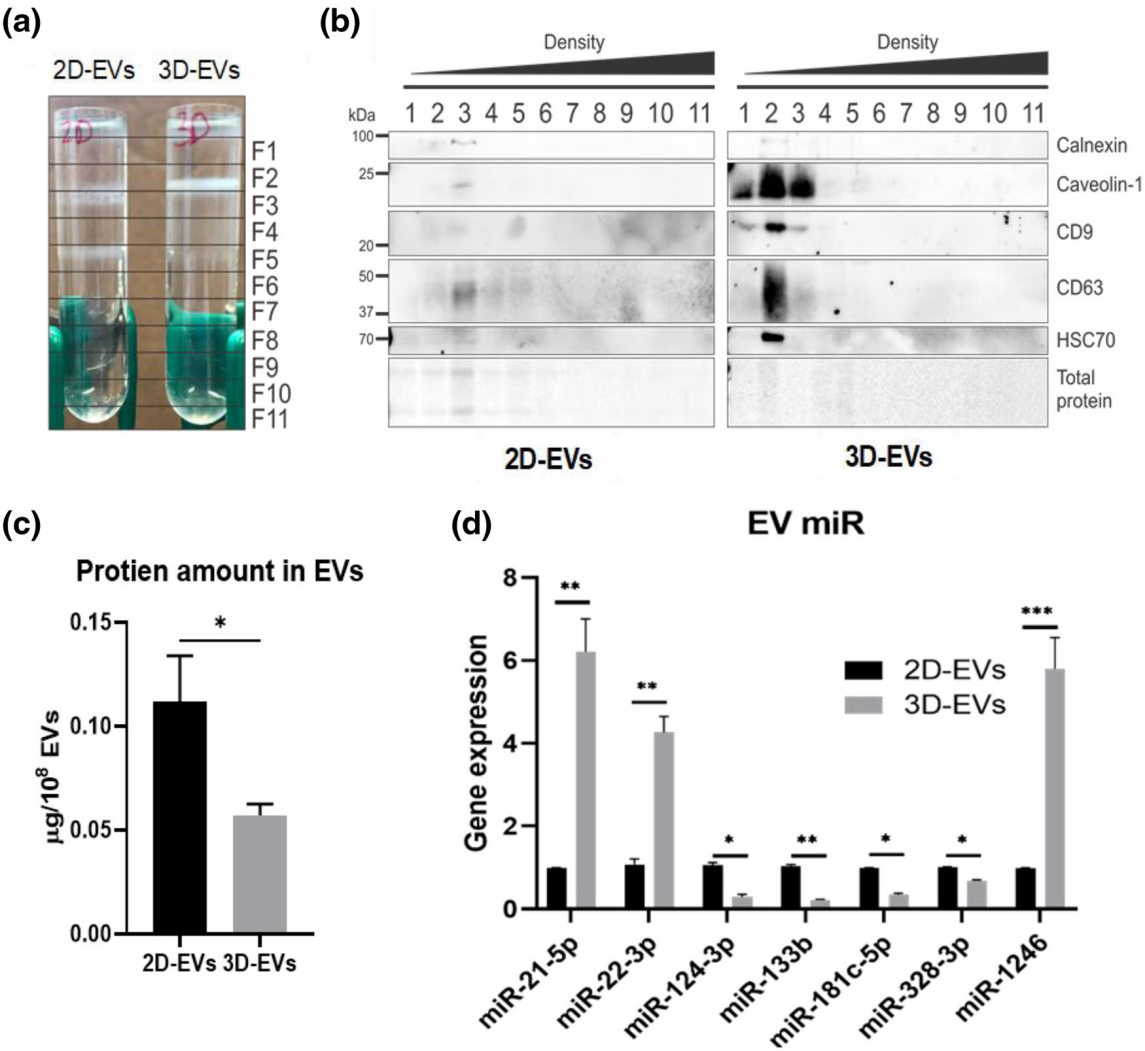
Protein and miRNA contents in EVs are altered by 3D dynamic culture. (a) Density gradient ultracentrifugation demonstrated enrichment of different sub‐populations between 2D‐ and 3D‐hMSC‐EVs. (b) Western blot analysis of EV markers in fractions from (a). (c) Total protein quantification in 2D‐ and 3D‐hMSC‐EVs (*n* = 3). (d) Expression of selected miRNAs in 2D‐ and 3D‐hMSC‐EVs determined by qRT‐PCR (*n* = 3). *P < 0.05; **P < 0.01; ***P < 0.001

Density gradient isolation was performed via ultracentrifugation, and eleven fractions were collected (Figure 4a and b). Fraction 2 and 3 are usually considered as exosome‐enriched fractions based on the density (Hurwitz & Meckes, 2017). In these results, more EVs in fraction 2 and 3 of 3D‐hMSC‐EVs clearly are observed (Figure 4a). The slightly higher density of fraction 2 EV band in 3D versus 2D conditions implies lighter density and lower amount of protein cargo packing. The EV markers of different fractions of 3D and 2D conditions were confirmed by Western blot (Figure 4b). Caveolin‐1, CD9, CD63 and HSC70 are significantly higher in fractions 2 and 3 of 3D‐hMSC‐EVs than other fractions, which is also higher than the corresponding 2D EVs. The absence of Calnexin in the EVs was confirmed. The total protein amount per 10^8^ EVs is also different between 2D and 3D‐hMSC‐EVs (∼0.12 vs. ∼0.05 μg; Figure 4c), which may be due to the smaller EV size in the 3D group (Figure 3e). miRNA cargo in the isolated 2D and 3D‐hMSC‐EVs was determined by qRT‐PCR (Figure 4d). The expression of miR‐21‐5p, miR‐1246 and miR‐22‐3p increases by 26‐fold, 5.2‐fold and 2.5‐fold, respectively, in 3D‐hMSC‐EVs compared to 2D‐hMSC‐EVs. Downregulation of miR‐124‐3p, miR‐133b, miR‐181c‐5p and miR‐328‐3p in 3D‐hMSC‐EVs is observed. Therefore, dynamic 3D aggregation culture significantly alters the expression of miRNAs with therapeutic relevance.

**FIGURE 5:**
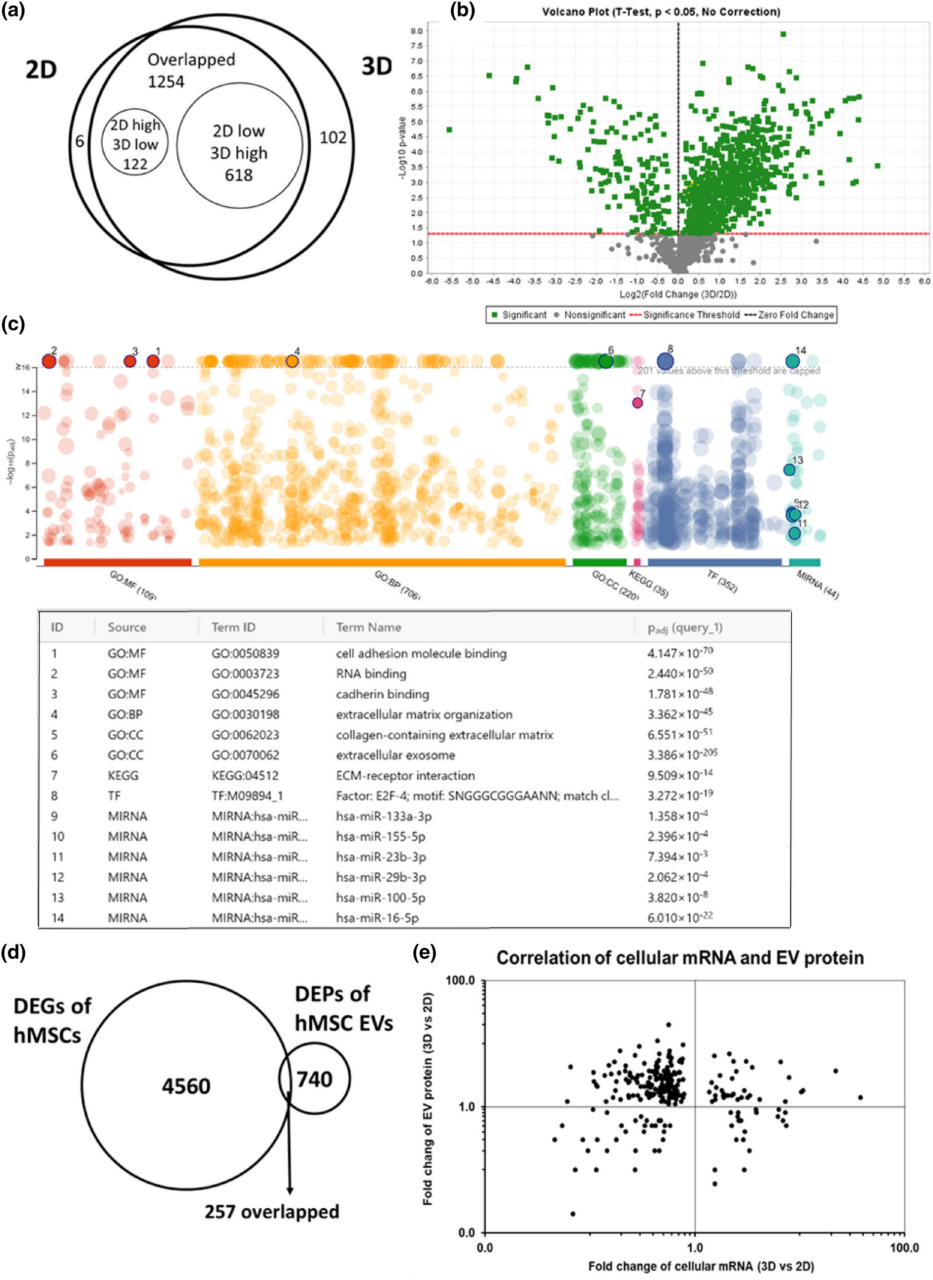
Proteomics analysis for 2D‐ and 3D‐hMSC‐EVs. (a) Venn diagram plot for 2D‐ and 3D‐hMSC‐EV along with differentially expressed proteins (DEPs). (b) Volcano plot of the DEPs, showing more DEPs were upregulated in 3D‐hMSC‐EVs. (c) Gene Ontology (GO) and Kyoto Encyclopaedia of Genes and Genomes (KEGG) analysis on the DEPs by g:Profiler. (d) The analysis to correlate DEGs in parent hMSC transcriptome with DEPs in the secreted hMSC‐EV protein cargo in order to explain the functional outcome of the hMSC‐EV recipient cells. Venn diagram of 4560 differentially expressed genes (DEGs) from hMSC mRNA‐sequencing with 740 DEPs from hMSC EV proteomics data identified 257 overlapped DEGs/DEPs. (e) Plot of fold‐change from 257 overlapped DEGs and DEPs as in (d)

To determine the specific protein cargo in the isolated EVs, proteomics by liquid chromatography with tandem mass spectroscopy (LC‐MS/MS) was performed for 2D‐ and 3D‐hMSC‐EVs (Figure 5). A Venn diagram plot shows a total of 1,254 shared proteins, 102 distinct proteins (7.5% in total proteins identified) for 3D‐hMSC‐EVs, and six distinct proteins for 2D‐hMSC‐EVs (Figure 5a and data file S3). Among the differential expressed proteins (DEPs), 122 (9.7%) are upregulated in 2D‐hMSC‐EVs, and 618 (49.3%) are upregulated in 3D‐hMSC‐EVs. A volcano plot of the proteomics data shows the DEP distribution in the expression levels for 3D‐ versus 2D‐hMSC‐EVs, and more DEPs were upregulated in the 3D‐hMSC‐EV group (Figure 5b). The top DEPs upregulated in 3D‐hMSC‐EVs are shown in data file S4. The top 20 enriched KEGG pathways from all DEPs are shown in Table 3, including focal adhesion, ECM‐receptor interactions, regulation of actin cytoskeleton and the PI3K‐Akt signalling pathway, which are correlated to the 3D aggregate culture system and RNA‐seq data from 3D hMSCs (Table 1). Gene Ontology (GO) annotation also reveals proteins from the extracellular space and matrix (Figure 5c, data files S5 and S6). In addition, pathways related to neurological disorders (e.g., Parkinson disease, Huntington disease, amyotrophic lateral sclerosis and spinocerebellar ataxia) are identified in GO analysis, which indicated potential therapeutic effects of hMSC‐EVs on neurodegenerative diseases

**TABLE 3:** The top 20 enriched KEGG pathways from DEPs between the proteomes of 3D‐hMSC‐EVs and 2D‐hMSC‐EVs

Pathway	ID	*P* value
Ribosome	KEGG:03010	4.26E‐21
Coronavirus disease ‐ COVID‐19	KEGG:05171	9.48E‐20
Proteasome	KEGG:03050	2.03E‐16
Focal adhesion	KEGG:04510	9.23E‐15
ECM‐receptor interaction	KEGG:04512	9.51E‐14
Protein processing in endoplasmic reticulum	KEGG:04141	1.29E‐08
Prion disease	KEGG:05020	3.45E‐08
Proteoglycans in cancer	KEGG:05205	6.18E‐08
Spinocerebellar ataxia	KEGG:05017	5.10E‐07
Salmonella infection	KEGG:05132	6.06E‐07
Phagosome	KEGG:04145	1.20E‐06
Parkinson disease	KEGG:05012	1.91E‐06
Regulation of actin cytoskeleton	KEGG:04810	3.38E‐06
Amoebiasis	KEGG:05146	7.78E‐06
Bacterial invasion of epithelial cells	KEGG:05100	2.86E‐05
Huntington disease	KEGG:05016	3.39E‐05
Protein digestion and absorption	KEGG:04974	4.98E‐05
Amyotrophic lateral sclerosis	KEGG:05014	4.99E‐05
PI3K‐Akt signalling pathway	KEGG:04151	5.29E‐05
Pathogenic Escherichia coli infection	KEGG:05130	9.14E‐05

Abbreviations: KEGG, Kyoto Encyclopaedia of Genes and Genomes; DEP, differentially expressed protein.

To understand the correlation between the transcriptome of parental hMSCs and the protein cargo profile of EVs revealed by proteomics data, the mRNA‐seq dataset (4560 DEGs) of hMSCs and the proteomics dataset (740 DEPs) of corresponding EVs were analysed together. There are 257 proteins/genes identified by both mRNA‐seq and proteomics analysis (Figure 5d). The mRNA (in hMSCs) and protein (in EVs) correlations did not show a linear relationship (Figure 5e and data file S7), indicating that the upregulated/downregulated expression of genes in the parental hMSCs do not necessarily lead to the high/low expression of proteins in the corresponding secreted EVs. This result further supports the hypothesis that specific mechanisms exist for EV cargo loading that are not simply a factor of intracellular protein expression levels.

### 2.4 Immunomodulatory potentials of 3D EVs and functional analysis in various in vitro models

**FIGURE 6:**
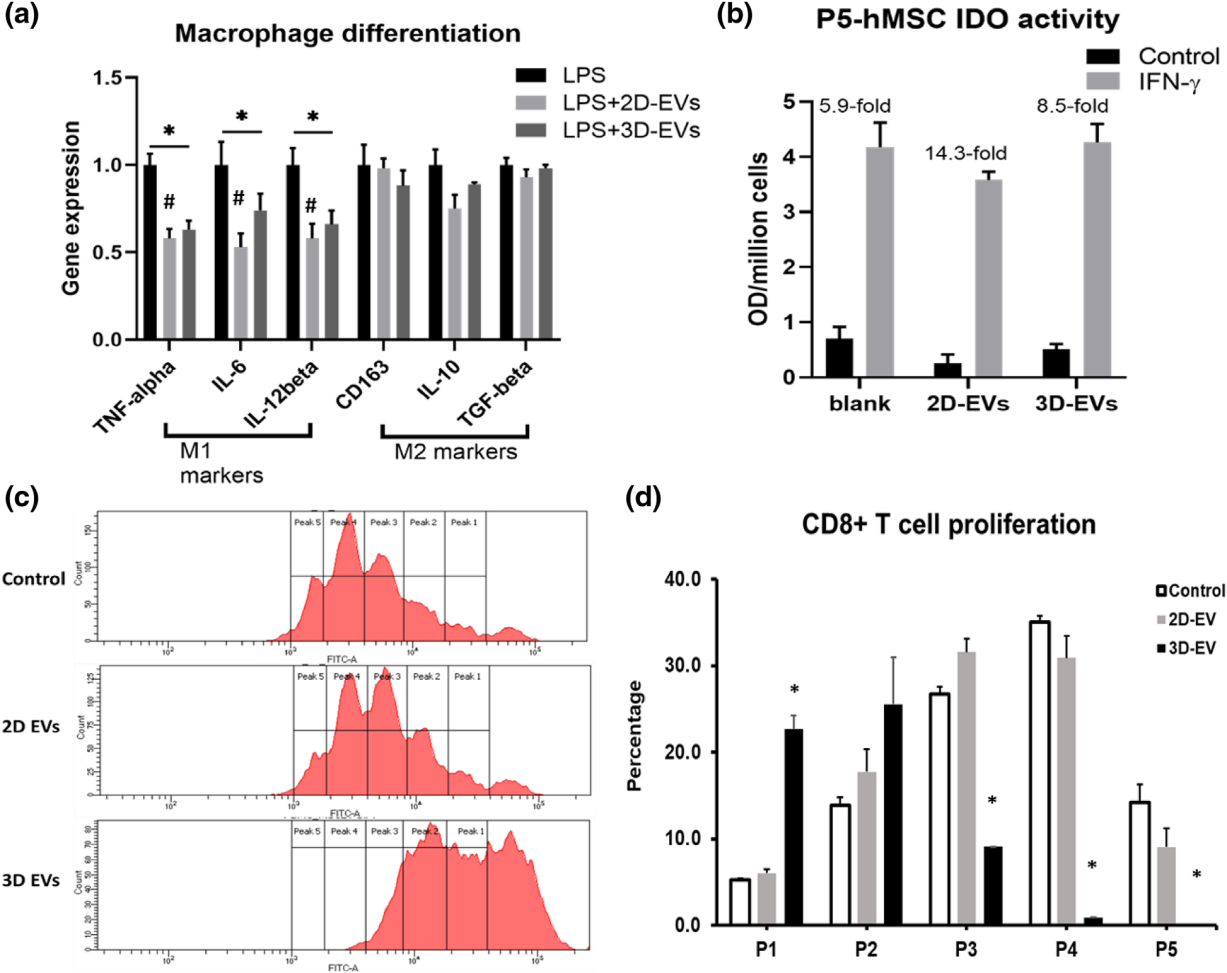
2D‐ and 3D‐hMSC‐EVs exhibit immunomodulatory properties. (a) M1 and M2 marker gene expression levels of macrophage upon stimulation with lipopolysaccharide (LPS), when treated with 2D‐ or 3D‐hMSC‐EVs (*n* = 3). (b) Indoleamine 2,3‐dioxygenase (IDO) activity from basal level and Interferon (IFN)‐γ priming of hMSCs (at P5) treated with EVs (*n* = 3). OD: optical density. (c) Representative flow cytometry histogram plot of CD8+ T cell proliferation profile from triplicate samples. (d) T cell proliferation peaks were measured in the bar graph for control, 2D‐hMSC‐EV, and 3D‐hMSC‐EV conditions (*n* = 3). *and #*P* < 0.05; **P < 0.01

Our previous study has established the M1/M2 macrophage polarization model for determining the immunomodulation properties of hMSCs (Jeske et al., 2021). In this study, the M0 phenotype and M1/M2 induction of the macrophage model were confirmed (Figure S7). The mRNA levels of pro‐ and anti‐inflammatory cytokines of macrophages were determined for the cells after lipopolysaccharide (LPS)‐stimulation (i.e., M1 pro‐inflammatory activation) together with 2D‐ and 3D‐hMSC‐EV treatment (Figure 6a). Both 2D‐ and 3D‐hMSC‐EVs reduced the mRNA level of pro‐inflammatory cytokines, *TNF‐α*, *IL‐6*, and *IL‐12β*, when compared to an untreated control (Figure 6a). In contrast, no effect was observed for the mRNA levels of anti‐inflammatory cytokines *CD163, IL‐10* and *TGF‐β*. However, for the EV‐treated M0 macrophages, no obvious M1 or M2 induction was observed (Figure S7). These results suggest that hMSC EVs potentially inhibit M1 (or favour M2) macrophage polarization and suppress pro‐inflammatory activity. No difference in macrophage polarization effect was observed between 2D‐ and 3D‐hMSC‐EVs.

To evaluate the immunomodulation ability of the isolated 3D‐hMSC‐EVs, indoleamine 2,3‐dioxygenase (IDO) activity was tested in hMSCs under interferon (IFN)‐γ stimulation. The change of IDO expression in hMSCs after 24‐h IFN‐γ licensing is 5.9‐fold in an untreated control compared to an 8.5‐fold increase by adding 3D‐hMSC‐EVs and 14.3‐fold for 2D‐hMSC‐EVs (Figure 6b). After IFN‐γ treatment, hMSCs were “licensed” to exert the immunomodulatory effect through the activation of IDO enzyme. Therefore, the fold‐change of IDO activity in hMSCs represents the potentials of the EVs to modulate local inflammatory environments. T cell proliferation assay was performed to evaluate immunosuppressive potentials of hMSC EVs. Compared to control, 2D‐hMSC‐EVs slightly inhibit CD8+ cytotoxic T cell proliferation, which can be observed by a major peak shift from P4 to P3/4 (Figure 6c and d). In contrast, 3D‐hMSC‐EVs (equal EV protein loading) result in significant reduction of total proliferating CD8+ T cells than the control sample. Since 3D‐hMSC‐EVs were smaller, equal protein loading may require higher EV number compared to 2D‐hMSC‐EV group, thus the EV dose effect was tested (Figure S8). The two different EV doses show similar effects, indicating that the dose range used in this study is appropriate. For CD4+ T helper cells, similar trend was observed compared to CD8+ cell inhibition (Figure S8). Together, these results indicated that hMSC EVs exert immunomodulatory potentials, and 2D‐ and 3D‐hMSC‐EVs may have different regulatory roles in various inflammation models.

**FIGURE 7:**
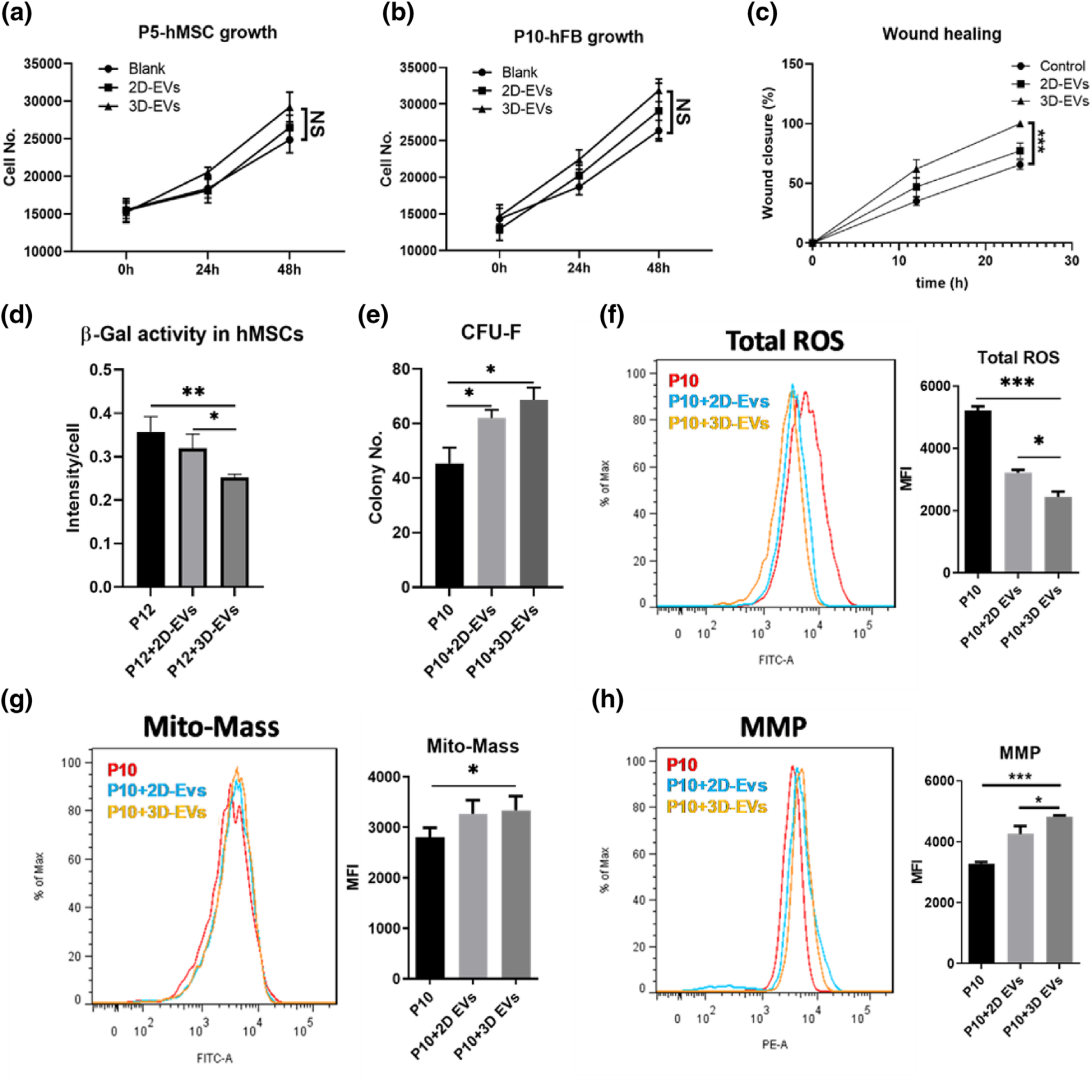
2D‐ and 3D‐hMSC‐EVs exhibit different functional outcome in stimulation of hFB expansion and rejuvenation of aged hMSCs with replicative senescence. (a) Cell number kinetics for hMSCs treated with 2D‐ or 3D‐hMSC‐EVs (*n* = 3). (b) Cell number kinetics for human fibroblast (hFB) treated with 2D‐ or 3D‐hMSC‐EVs (*n* = 3). (c) Wound closure percentage of hFBs treated with 2D‐ or 3D‐hMSC‐EVs (*n* = 3). (d) β‐Gal activity of senescent hMSCs (P12) with EVs (*n* = 3). (e) Colony‐forming unit‐fibroblast (CFU‐F) numbers of senescent hMSCs (P10) (*n* = 3). (f) Total reactive oxygen species (ROS), (g) Mitochondrial mass (Mito‐Mass) and (h) Mitochondria membrane potential (MMP) levels were determined by flow cytometry for senescent hMSCs (P10) treated by EVs (*n* = 3). *P < 0.05; **P < 0.01; ***P < 0.001. NS: not significant

As the population and internal cargo were significantly altered by 3D aggregation culture (Figures 3, 4, 5), the therapeutic potentials of 3D‐hMSC‐EVs were evaluated in several in vitro culture models. Both 2D‐ and 3D‐hMSC‐EVs were added as a culture supplement during hMSC and human fibroblast (hFB) expansion. Cell proliferation was promoted in both cell types compared to phosphate‐buffered saline (PBS) control. While 3D‐hMSC‐EVs tend to better enhance cell growth, no significance is observed compared to 2D‐hMSC‐EVs (Figure 7a and b). Similarly, in an in vitro wound healing model (scratch assay), 3D‐hMSC‐EVs promote faster wound closure compared to 2D‐hMSC‐EVs (Figure 7c). When hMSCs were expanded to higher passage (P10–P12), cells exhibit replicative senescence as shown previously (Yuan, 2020). In this in vitro cellular aging model, by adding 3D‐hMSC‐EVs as a culture supplement, cellular senescence is significantly reduced indicated by lower β‐Gal activity in P12 hMSCs compared to a 2D‐hMSC‐EV‐treated group (Figure 7d). Moreover, both 2D‐ and 3D‐hMSC‐EVs significantly increase self‐renewal potentials of P10‐senescent hMSCs, demonstrated by increased CFU‐F number (Figure 7e). The total reactive oxygen species (ROS) levels in senescent hMSCs (P10)  also are reduced more significantly by 3D‐hMSC‐EVs compared to 2D‐hMSC‐EVs (Figure 7f). In addition, 3D‐hMSC‐EVs helped to maintain mitochondrial fitness in senescent hMSCs (P10), indicated by the increased membrane potential (MMP) level after EV treatment (Figure 7g and h). These results reveal a potential anti‐aging/anti‐senescence effect of hMSC‐EVs especially for the EVs generated by 3D dynamic aggregation culture.

To further interpret the functional results (Figures 6 and 7), potential candidates were proposed from the integrated analysis of multi‐omics data (parental hMSC mRNA‐seq and EV proteomics). Several DEGs and DEPs were classified into different categories and matched results from the functional assays (Table 4). First, it was noticed that there is less correlation between transcriptome and proteome data, which may imply a distinct EV protein cargo sorting mechanism in 3D hMSCs. For EV production, protein markers (e.g., VIM, CD81 and CD63) were only identified in proteomics data (verified by Western blot in Figure 3c), but the EV secretion component Rab27B was only found in cellular transcriptome (confirmed by qRT‐PCR in Figure 2d). The increased levels of miRNA‐21 may explain the anti‐inflammation effects on macrophage polarization (Figures 4d and 6a), but it could also be attributed to overexpressed low density lipoproteins (LDL), for example, APOE and HDLBP, that reduce the effect of LPS (Table 4). There are several candidates correlated with T cell suppression, but the more compelling evidence comes from the mRNA expression levels in 3D hMSCs. How these EV proteins affect T cell phenotype and molecular biology needs to be further investigated. This list also may be incomplete due to complex indirect effects such as activation of myeloid‐derived suppressor cells (MDSCs) in peripheral blood mononuclear cells (PBMCs). Several cytokines and other secreted soluble factors (e.g., FAP, TGFB, VEGFA, PLAU and SERPINE1) were identified that are related to wound healing and cell proliferation (Figure 7a‐c). The individual effect and/or contribution of each cytokine requires further investigations. ROS scavenging proteins were all upregulated in either the cells (by mRNA‐seq) or EVs (by proteomics), which could be a potential mechanism for the observations in Figure 7f‐h. Collectively, hMSC EVs possess therapeutic cargo contents for various functions and the culture strategy of 3D aggregation of hMSCs significantly impacts EV secretion and cargo profile.

**TABLE 4:** The identified DEGs (mRNA‐seq) and DEPs (proteomics) between 3D and 2D conditions for hMSCs and EVs, respectively, related to functional assays

Function	Gene symbol	Dataset	*P* value	Fold change^a^
Reduce LPS induction of M1	HDLBP	Transcriptome	1.3E‐02	0.8
		Proteome	1.1E‐03	2.7
	APOE	Transcriptome	9.5E‐40	37.8
		Proteome	1.6E‐03	1.4
Inhibit T cell proliferation	CD274	Transcriptome	8.0E‐09	0.2
	CHI3L1	Transcriptome	2.8E‐07	20.3
	CD82	Transcriptome	3.9E‐10	7.9
		Proteome	< 0.00010	2.9
	NPTN	Transcriptome	1.4E‐02	0.7
		Proteome	< 0.00010	3.5
	EZR	Transcriptome	2.1E‐110	0.1
		Proteome	6.2E‐04	2.5
	MSN	Transcriptome	5.3E‐09	0.5
		Proteome	6.1E‐04	1.9
	CD248	Transcriptome	3.2E‐06	0.2
		Proteome	4.3E‐02	1.3
	HMGB1	Transcriptome	1.0E‐02	0.6
		Proteome	1.9E‐02	1.5
EV marker, Inhibit T cell proliferation	CD81	Proteome	1.3E‐03	1.8
Exosome secretion	RAB27B	Transcriptome	1.1E‐22	10.1
EV marker	VIM	Transcriptome	1.5E‐12	0.2
		Proteome	< 0.00010	3.7
	CD63	Proteome	< 0.00010	2.1
Wound healing and angiogenesis	FAP	Transcriptome	1.8E‐06	3.1
		Proteome	1.4E‐04	5.2
	PDGFC	Transcriptome	3.5E‐03	0.6
		Proteome	NS*	
	NCL	Transcriptome	1.2E‐04	0.6
		Proteome	3.7E‐04	6.2
	MYH9	Transcriptome	1.5E‐20	0.3
		Proteome	NS	1.1
	ROCK2	Transcriptome	4.1E‐03	0.4
		Proteome	1.8E‐03	1.7
	YWHAZ	Transcriptome	3.8E‐05	0.5
		Proteome	3.6E‐03	1.7
	CTNNB1	Transcriptome	2.5E‐04	1.7
		Proteome	9.7E‐03	3.1
	TGFB1	Transcriptome	1.7E‐04	3.0
		Proteome	2.9E‐03	0.4
	TGFB2	Transcriptome	1.7E‐03	2.2
		Proteome	1.4E‐03	0.5
	ANGPT1	Transcriptome	3.3E‐04	0.4
		Proteome	< 0.00010	0.5
	PLAU	Transcriptome	6.9E‐06	2.7
		Proteome	NS	
	SERPINE1	Transcriptome	1.0E‐15	0.2
		Proteome	5.6E‐04	0.3
	VEGFA	Transcriptome	2.4E‐09	5.4
Mitochondria function and ROS scavenging	GPX3	Transcriptome	5.1E‐03	2.8
		Proteome	2.8E‐02	1.4
	GPX7	Proteome	NS	
	PRDX1	Proteome	< 0.00010	3.0
	PRDX2	Transcriptome	1.5E‐02	1.9
		Proteome	1.6E‐02	1.6
	PRDX6	Transcriptome	2.9E‐02	0.8
		Proteome	2.3E‐02	1.5
	IDH1	Proteome	5.5E‐04	2.5
	SOD2	Transcriptome	1.5E‐13	12.1
	NRROS	Transcriptome	2.0E‐12	14.2

* NS: not significant.

^a^ Upregulated shown in red and downregulated shown in green.

## 3 DISCUSSION

Previous studies have demonstrated the beneficial effects of 3D aggregation culture of hMSCs in retaining the primitive stem cell phenotype, reducing the cellular senescence and therapeutic benefits in animal models of ischemic stroke (Bijonowski et al., 2020; Bijonowski et al., 2019; Sart et al., 2014; Tsai et al., 2017; Tsai et al., 2015; Yuan et al., 2019). While the effects of 3D aggregation of hMSCs on the secretome composition (i.e., the secreted growth factors or cytokines) has been investigated (Bartosh et al., 2010; Liu et al., 2017), the potential influence on the hMSC EV biogenesis and functional outcomes of the hMSC aggregate secreted EVs have not been well studied.

In this study, enhanced EV production, reduced EV size (indicating the enriched exosome population) and increased expression of exosomal markers CD63 were observed for EVs secreted by hMSCs under dynamic 3D aggregation culture. While the per EV‐based protein content was lower for the 3D group than the 2D group, given the higher EV yield of 3D culture, the total EV protein content was comparable for 3D‐hMSC‐EVs and 2D‐hMSC‐EVs. Some of the endosomal proteins in hMSCs (e.g., Rab27B, determined by qRT‐PCR and among the DEGs identified by mRNA‐Seq) may be responsible for the increased exosome secretion and functional outcomes of the 3D EVs in recipient cells. Both ESCRT‐dependent and ESCRT‐independent EV biogenesis markers were upregulated in 3D hMSC aggregates, which may have contributed to enhanced EV production and cargo sorting (Colombo et al., 2014; Stuffers et al., 2009). The smaller EV size may explain partially the high CD63 expression in 3D‐hMSC‐EVs as CD63 is present on the small EV subpopulation (Kowal et al., 2016). 3D culture has been reported to be more efficient in generating EVs than 2D culture for gastric cancer cells (Haraszti et al., 2018; Rocha et al., 2019), probably due to cytoskeleton reorganization as well as the global upregulation of miRNAs (Liu & Su, 2019; Rocha et al., 2019). Consistently in the current mRNA‐seq results, a majority of downregulated cytoskeleton genes in 3D hMSC aggregates is evident, which may explain the enhanced EV production. In addition, cytoskeletal blocking using a sulfhydryl‐blocking agent, hindering cytoskeletal function using mercapto blockers, and using actin/myosin inhibitors have been reported to promote EV production (Liu & Su, 2019; Zaborowski et al., 2015).

mRNA sequencing of the transcriptome of 3D hMSC aggregates in this study showed the upregulation of Wnt, TNF, Hippo and MAPK signalling pathways and the downregulation of cell cycle, DNA replication and cellular senescence associated pathways. 3D culture has been found to promote cell‐cell interactions (e.g., Notch pathway), ECM secretion and enrichment for various cell types (Bejoy et al., 2019; Song et al., 2019; Wrzesinski & Fey, 2018). hMSCs in 3D aggregates do not proliferate well as they are in cell cycle arrest, confirmed by the downregulation of cell cycle‐related genes. However, cellular senescence is reduced in 3D hMSC aggregates due to better preservation of quiescent stem cells as demonstrated previously (Bijonowski et al., 2020). There is no necrotic or hypoxic centre in 3D hMSC aggregates (< 500 μm diameter), which can occur in other 3D structures due to diffusion limitations (Bijonowski et al., 2019). Moreover, previous work has shown that hMSCs shift their metabolic pathway towards glycolysis, which adapts them to the 3D microenvironment and supports a reduced requirement for oxygen uptake; thus, oxygen in hMSC aggregates under a certain diameter is not diffusion‐limited (Bijonowski et al., 2019; Liu et al., 2017; Liu et al., 2019). The metabolic reconfiguration and stress‐response in 3D hMSC aggregates likely induce the activation of a broad signalling pathways, including apoptosis characterized by increased caspase 3/7 activity, which also may contribute to the increased EV production (Bijonowski et al., 2020; Liu et al., 2017; Tsai et al., 2015; Yuan et al., 2019). Moreover, apoptotic bodies can be removed by the ExtraPEG purification, and thus unlikely to be the therapeutic population of 3D‐hMSC‐EVs. Therefore, the effects of 3D aggregation on hMSC EV biogenesis likely result more from enhanced cell‐cell interactions and ECM enrichment, as well as associated intracellular signalling pathways.

This study reveals the significantly increased expression of neuroprotective and anti‐apoptotic miRNAs in the 3D‐hMSC‐EVs compared to the 2D condition. These miRNAs play various beneficial roles in immunomodulation and neural protection (Rocha et al., 2019). For example, downregulated miR‐133b contributes to the inhibition of macrophage activation (Zheng et al., 2019); upregulated miR‐1246 is associated with macrophage reprograming, stress response and possible angiogenesis (Cooks et al., 2018; Irizar et al., 2015). miR‐21‐5p was found to be upregulated in remission periods of multiple sclerosis (Baulina et al., 2018); miR‐22 was reported to regulate immune response via p38 and MAPK pathway (Krementsov et al., 2013). In addition, miR‐21 shows potent neuroprotective effects after spinal cord injury, alleviates blood‐brain barrier disruption in ischemic stroke, and promotes neurite outgrowth by regulating programmed cell death protein 4 (PDCD4) (Jiang et al., 2017; Li et al., 2012; Yao et al., 2018; Zhang et al., 2019). For Alzheimer's disease, miR‐21 attenuates neural inflammation, and miR‐124 regulates beta‐site amyloid precursor protein (APP) cleaving enzyme 1 (BACE1) (Amakiri et al., 2019; Jeske et al., 2020; Reddy et al., 2017). miR‐328 and miR‐181 were downregulated in Alzheimer's pathology (Amakiri et al., 2019). miR‐22 attenuates neuronal cell apoptosis, and controls neuronal migration and neurogenesis (Berenguer et al., 2013; Ma et al., 2016; Volvert et al., 2014). The beneficial miRNA alterations (e.g., miR‐21) may contribute to the functional improvements in preclinical models, and require further investigations. Indeed, current reports with preclinical applications of 3D‐hMSC‐EVs are very limited. Our previous study showed that 5XFAD (5 familial Alzheimer's disease mutations) mice that received 3D‐hMSC‐EVs behaved significantly better in cognitive tests than saline control (Cone et al., 2021). In addition, lower Aβ plaque load was observed in the hippocampus of the EV‐treated mice, and less co‐localization of GFAP and Aβ plaques was found in the brains of EV‐treated mice compared to saline (Cone et al., 2021).

Proteomics have been used to identify the novel protein markers for sub‐population of hMSC EVs, such as the discovery of adhesion protein enrichment in small EVs, as well as the proteins associated with anti‐inflammatory ability (Jimenez et al., 2019; Kowal et al., 2016; Upadhya et al., 2020). The current proteomics results reveal the upregulation of 618 (49.3%) DEPs and 102 (7.5%) proteins uniquely expressed in 3D‐hMSC‐EVs compared to the 2D condition, which are related to focal adhesion, ECM‐receptor interactions, regulation of actin cytoskeleton, and PI3K‐Akt signalling pathway. The 3D‐hMSC‐EV protein cargo also may be responsible for promoting cell viability, migration, T cell inhibition, macrophage polarization/function and axon/neuron development. Compared to the mRNA‐seq results, the upregulated/downregulated gene expression in the parental hMSCs does not necessarily lead to high/low protein expression in the secreted EVs, indicating that there may be specific sorting mechanisms of EV cargo. The overlaps of DEGs and DEPs were found to be related to anti‐inflammatory/immunomodulatory abilities, including neutrophil and other myeloid leukocyte mediated immunity and immune response, T cell receptor signalling pathway, and neurotrophic action. The effects of the protein cargo in 3D‐hMSC‐EVs were verified by various functional analyses in this study.

To evaluate the immunomodulatory effect, macrophages treated with 2D‐ and 3D‐hMSC‐EVs during polarization were found to inhibit the M1 differentiation at the molecular level. Notably, mRNA‐seq analysis showed the upregulation of IL10 and TGFB1 for 3D hMSCs. However, their protein levels in cell conditioned media were not detectable. In addition, during the EV isolation (ExtraPEG), PBS wash was performed in the last ultracentrifugation step, which may remove soluble cytokines. In the proteomic results, only TGFB1 and TGFB2 were detected (no IL‐10), which were lower in 3D‐hMSC‐EVs (0.5 fold). Therefore, the effects of EVs on macrophage polarization may not be due to IL10 and TGFB1. Nonetheless, the cytokine secretion by polarized macrophages may need to be investigated in future. The immunosuppressive properties of hMSC‐derived exosomes have been reported previously (Cosenza et al., 2018). Both 2D‐ and 3D‐hMSC‐EVs were found to enhance the immunomodulation potentials of recipient hMSCs characterized by IDO activity, which is a major signalling cascade for hMSC immunomodulation under inflammation (Liu et al., 2019; Ren et al., 2009). These results provide the possibility for using hMSC‐EVs as supportive agents to improve therapeutic outcomes after cell transplantation. In addition, both 2D‐ and 3D‐hMSC‐EVs have shown significant inhibition of cytotoxic T cell proliferation, and the EV effects on helper T cells are similar to cytotoxic T cells. The expression of chitinase 3 like 1 protein (encoded by CHI3L1, increase by 4.34‐fold based on mRNA‐seq results) likely contributes to the immunomodulation of hMSC EVs on the T cells (Table 4) (Liu et al., 2021).

Moreover, 3D‐hMSC‐EVs exerted anti‐senescence ability to maintain cellular homeostasis and mitochondrial fitness in recipient senescent hMSCs due to in vitro replication. The total ROS levels in senescent hMSCs were reduced significantly by adding 3D‐hMSC‐EVs in culture media as supplement. Consistently, hMSC‐derived EVs have been reported to reduce oxidative stress and increase ATP levels (Arslan et al., 2013). Therefore, hMSC EVs could be added as culture supplement to rejuvenate adult stem cells or to reconfigure central energy metabolism of hMSCs in biomanufacturing (Yuan, 2020). 3D‐hMSC‐EVs also promote fibroblast growth and wound healing in vitro, probably due to the upregulated VEGFA and FAP expression (Table 4). These features motivate the use of 3D‐hMSC‐EVs with targeted miRNA and protein payloads for multiple sclerosis, ischemic stroke and Alzheimer's disease therapies, as well as retaining endogenous tissue homeostasis. Moreover, considering that 3D aggregation culture of hMSCs promoted the yield of the secreted EVs with comparable or better functional outcomes than the 2D condition, the scalable 3D aggregation in bioreactors greatly expands the production potential of therapeutic EVs from hMSCs for translational research and biomanufacturing.

There are some limitations in current study: for instance, macrophage polarization is solely based on the mRNA expression of specific markers rather than the cytokine secretion. The actual large‐scale production (e.g., in a full scale of wave bioreactor or vertical wheel bioreactor) of 3D‐hMSC‐EVs for preclinical studies has not been fully demonstrated. The translational potential is highly dependent on the large‐scale culture system for 3D hMSC aggregates and the secreted EVs in the bioreactors need to be thoroughly characterized. The consistency of the protein and miRNA cargo of 3D‐hMSC‐EVs for each bioreactor run also needs to be demonstrated for quality control. The follow‐up study should identify the key contributing cargo (e.g., the specific protein or miRNA) in the 3D‐hMSC‐EVs and further engineer 3D‐hMSC‐EVs to maximize in vivo functional/therapeutic outcomes.

## 4 MATERIALS AND METHODS

### 4.1 hMSC standard 2D culture

Frozen hMSCs from passage 0 to 2 were acquired from Tulane Centre for Gene Therapy. The hMSCs were isolated from the bone marrow of multiple healthy donors with age 19–49 years old (Table S3) based on plastic adherence, negative for CD3, CD14, CD31, CD45 and CD117 (all less than 2%) and positive for CD73, CD90, CD105 and CD147 markers (all greater than 95%), and possess tri‐lineage differentiation potential upon in vitro induction. hMSCs (1 × 10^6^ cells/ml/vial) were cryopreserved in freezing media containing α‐MEM, 2 mM L‐glutamine, 30% foetal bovine serum (FBS) and 5% dimethyl sulfoxide (DMSO). hMSCs were thawed, expanded, and maintained in complete culture media (CCM) containing α‐MEM with 10% FBS (Atlanta Biologicals, Lawrenceville, GA) and 1% Penicillin/Streptomycin (Life Technologies, Carlsbad, CA) in a standard incubator at 37°C with 5% CO_2_ and 20% O_2_. Culture medium was changed every 3 days. Cells were grown to 70%‐80% confluence and then harvested by incubation with 0.25% trypsin/ethylenediaminetetraacetic acid (EDTA) (Invitrogen, Grand Island, NY, USA) at 37°C for 4–7 min. Harvested cells were re‐plated at a density of 1,500 cells/cm^2^ and sub‐cultured up to passage 4–6. For senescent hMSCs, the cells at passage 10–12 were used in the experiments.

### 4.2 3D dynamic culture of hMSCs as aggregates

hMSC 3D dynamic culture as aggregates was performed as reported in our previous studies (Bijonowski et al., 2020; Tsai et al., 2017; Yuan et al., 2019). Briefly, hMSCs at passage 4 to 7 were suspended in CCM and 1.0 × 10^5^ hMSCs in 2 mL CCM were seeded in each well of ultra‐low attachment (ULA) six‐well plates (Corning). The ULA plates were placed on a rocking base of a WAVE Bioreactor (Xuri Cell Expansion System W5, GE Healthcare) in a standard humidified incubator (37°C, 5% CO_2_) under controlled rocking angles (i.e., 8°) and rocking speeds (i.e., 20 rockings/min, or rpm) for 18 to 72 h. The rocking conditions generate wave motion of media under defined shear stress that supports spontaneous aggregation of hMSCs (100‐300 μm in diameter) (Sart et al., 2014). For 3D‐hMSC‐EV collection, the CCM was replaced by the media that contain EV‐depleted FBS (i.e., FBS was ultracentrifuged at 100,000 g for 18 h to remove particle and EVs) and cultured for 2 days before media collection. The parallel 2D hMSC culture was used as a control condition, and the conditioned media were collected after 2‐day culture for 2D‐hMSC‐EV isolation.

### 4.3 β‐Gal activity, cell number, and CFU‐F measurements

Cellular senescence was evaluated by a SA‐β‐Gal activity assay kit (Sigma, St. Louis, MO, USA) as described in manufacturer's instructions. Cell number was determined by Quant‐iT PicoGreen kit (Invitrogen). Briefly, cells were harvested, lysed overnight using proteinase K (VWR, Radnor, PA, USA), and stained with PicoGreen to allow quantitation of cellular DNA. Fluorescence signals were measured using a BioTek fluorescence plate reader (Agilent, Santa Clara, CA, USA). For CFU‐F assay, hMSCs were harvested and re‐plated at the density of 15 cells/cm^2^ on 60 cm^2^ culture dishes and grew for another 14 days in CCM. Cells were stained with 20% crystal violet solution in methanol for 15 min at room temperature and gently washed with phosphate‐buffered saline (PBS) three times. The number of individual colonies were counted manually.

### 4.4 Extracellular vesicle isolation and density gradient ultracentrifugation

Extracellular vesicles were isolated from the conditioned media by polyethylene glycol (PEG) precipitation and ultracentrifugation according to previous publications (Hurwitz et al., 2018; Rider et al., 2016). Briefly, cells were cultured in CCM with 10% EV‐depleted FBS, and the conditioned media were collected every 48 h. The conditioned media were differential centrifuged (500 g for 5 min; 2,000 g for 10 min; 10,000 g for 30 min) to remove larger debris, apoptotic body and microvesicles. Supernatants then were mixed with PEG solution (16% wt./vol. PEG‐6000, 1.0 M NaCl) at a 1:1 volume and incubated at 4°C overnight. The next day, the mixed solutions were centrifuged at 3,214 g for 1 h to obtain crude EVs. The pellets were resuspended in PBS and then ultracentrifuged at 100,000 g for 70 min using the SW‐28 swing‐bucket rotor in an Optima XL‐100K ultracentrifuge (k factor‐15, Beckman Coulter Inc.). The concentrated vesicle suspension was centrifuged again in 1 mL polypropylene tubes (Beckman Coulter Inc., #347287) in an Optima MAX‐E tabletop ultracentrifuge using a TLA120.2 rotor (Beckman Coulter Inc.) to purify the vesicles and facilitate re‐suspension of the pellet in a small volume of particle‐free PBS in 100 μL. Purified EVs were stored in −80°C for further use.

For sub‐population profiling by density gradient ultracentrifugation, EVs were resuspended in 1.5 mL of 0.25 M sucrose buffer (10‐mM Tris, pH 7.4). Gradients (10‐30%) were constructed as previously described in other studies (Hurwitz et al., 2019; Hurwitz & Meckes, 2017; Hurwitz et al., 2018; Kowal et al., 2016). Following fractionation, densities of gradient separated fractions were estimated by measuring refractive indices of fractions with a refractometer (Refracto 30PX, Refracto Technologies Corp., Bohemia, New York). Samples were then washed in 10 mL PBS and pelleted again by ultracentrifugation at 100,000 g for 2 h at 4°C. Pellets were resuspended in particle‐free PBS or lysis buffer for immunoblot analysis.

### 4.5 Nanoparticle tracking analysis (NTA)

NTA was performed on the isolated EV samples in triplicate to determine size distribution and particle concentration. NTA was performed on a NanoSight LM10‐HS instrument (Malvern Instruments, Malvern, UK) configured with a blue (488 nm) laser and CMOS camera (Rider et al., 2016). For each replicate, three videos of 60 s were acquired with camera shutter speed fixed at 30 ms. To ensure accurate and consistent detection of small particles, camera level was set to 13, and detection threshold was maintained at 5. The laser chamber was cleaned thoroughly with particle‐free water between each sample reading. The collected videos were analysed using NTA3.4 software to obtain the mode and mean size distribution, as well as the concentration of particles per mL of solution. The EV production was reported as the number of EVs normalized to the cell number at day 2, when the media were collected for EV isolation.

### 4.6 Transmission electron microscopy (TEM)

Electron microscopy imaging was performed to confirm the morphology and size distribution of EVs as shown previously (Marzano et al., 2019; Marzano et al., 2021). Briefly, first, intact EVs (5 μL) were dropped onto Parafilm. A carbon coated 400 Hex Mesh Copper grid (Electron Microscopy Sciences (EMS), Hatfield, PA, USA) was positioned using forceps with coating side down on top of each drop for 1 h. Grids were washed with sterile filtered PBS three times, and then the EV samples were fixed for 10 min in 2% PFA (EMS, EM Grade). After washing, the grids were transferred on top of a 20‐μL drop of 2.5% glutaraldehyde (EMS, EM Grade) and incubated for 10 min at room temperature. Grid samples were stained for 10 min with 2% uranyl acetate (EMS, EMS grade). Then the samples were embedded for 10 min with 0.13% methyl cellulose and 0.4% uranyl acetate. The coated side of the grids were left to dry before imaging on the CM120 Biotwin electron microscope (Field Electron and Ion Company, FEI, Hillsboro, OR, USA) (Lasser et al., 2012). Image analysis was performed in ImageJ to determine the average EV size and size distribution.

### 4.7 Western blot for EV markers

EV pellets following ultracentrifugation were lysed in 2× Laemmli sample buffer (4% SDS, 100 mM Tris‐HCl [pH 6.8], 0.4‐mg/ml bromophenol blue, 20% glycerol) for immunoblot analysis. The supernatant was collected, and a Bradford assay was carried out to determine the protein concentration. Protein lysate concentration was normalized, and 20 μg of each sample was denatured at 95°C in Laemmli sample buffer with 2‐mercaptoethanol. Proteins were separated by 15% Bis‐Tris SDS‐PAGE gel and transferred onto a nitrocellulose membrane (Bio‐Rad, Hercules, CA, USA). The membranes were blocked for 30 min in 5% skim milk power (w/v) in Tris‐buffered saline (10 mM Tris‐HCl [pH 7.5], and 150 mM NaCl) with 0.1% Tween 20 (v/v) (TBS‐T), or in 5% bovine serum albumin in TBS‐T. Membranes were incubated overnight in the presence of the primary antibodies (Table S4) diluted in the corresponding blocking buffer at 4°C (Dilution was shown in Table S3). Afterward, the membranes were washed four times for 10 min each with TBS‐T and then incubated with horseradish peroxidase (HRP)‐conjugated secondary antibodies: rabbit anti‐mouse IgG (Genetex, Irvine, CA, USA; 26728) or goat anti‐rabbit IgG (Fab fragment) (Genetex; 27171) for 1 h at room temperature. After wash with TBS‐T, the blots were incubated with SuperSignal West Pico Chemiluminescent Substrate (ThermoFisher Scientific, Waltham, MA, USA; 34080) and imaged using an ImageQuant LAS 4000 (GE Healthcare Bio‐Sciences Corp. Piscatoway, NJ, USA) and processed with ImageQuant TL v8.1.0.0 software.

### 4.8 Real‐time quantitative reverse transcription – polymerase chain reactions (qRT‐PCR)

For miRNA quantification, total RNA was isolated from different EV and cell samples using the miRNeasy Micro Kit (Qiagen, Valencia, CA, USA) according to the manufacturer's protocol. Reverse transcription was carried out using a commercial qScript miR cDNA synthesis kit (Quantabio, Beverly, MA, USA). The qPCR primer (along with a universal reverse primer) for each miRNA has been designed and validated to work specifically with miRNA cDNA reverse transcribed using the kit. The levels of miRNAs were determined, and SNORD44 was used as a housekeeping gene for normalization (Primer sequences are shown in Table S5). qPCR reactions were performed on a QuantStudio 7 Flex Real‐time PCR System (Applied Biosystems, Foster City, CA) using SYBR Green PCR Master Mix. The amplification protocol was performed as follows: 10 min at 95°C, and 40 cycles of 95°C for 15 sec and 60°C for 30 sec, and 70°C for 30 sec. Fold variation in gene expression was quantified by means of the comparative Ct method: $2^{-(\Delta C_{t\;\text{treatment}}-\Delta C_{t\;\text{control}})}$, which is based on the comparison of expression of the target gene (normalized to the endogenous control SNORD44) between the compared samples.

For mRNA quantification, total RNA was isolated using the RNeasy Plus kit (Qiagen) following vendor's instructions. Reverse transcription was carried out using 2.0 μg of total RNA, anchored oligo‐dT primers (Operon) and Superscript III (Invitrogen). Primers for specific target genes were designed using the software Oligo Explorer 1.2 (Genelink; Table S6). qPCR reactions were performed on an ABI7500 instrument (Applied Biosystems) using SYBR Green PCR Master Mix. The amplification reactions were performed, and the quality and primer specificity were verified. Fold variations in gene expressions were quantified using the comparative Ct method:$\;2^{-(\Delta C_{t\;\text{treatment}}-\Delta C_{t\;\text{control}})}$, which is based on the comparison of the target gene (normalised to endogenous gene) among different conditions.

### 4.9 Cellular mRNA next generation sequencing and data analysis

Total RNA was extracted from various samples using Trizol (ThermoFisher Scientific, 15596026). mRNA was isolated from the total RNA using an NEBNext Poly(A) mRNA Magnetic Isolation Module (New England Biolabs (NEB), Ipswich, MA, USA, E7490). cDNA libraries were generated from the isolated mRNA using an NEBNext Ultra RNA library prep kit for Illumina (NEB, E7530), and a unique six nucleotide index primer was incorporated into each sample. The library construction was done according to the NEB manuals. The multiplexed sample was quantified with KAPA qPCR (Kapa Biosystems, Potters Bar, UK, KR0405) specific for Illumina sequencing primers and the average fragment size was determined with a Bioanalyzer high sensitivity DNA chip (Agilent Technologies, Santa Clara, CA, USA, 5067‐4626). Pooled sample was sequenced with single end, 100 base reads on a NovaSeq 6000 system (Illumina, Inc., San Diego, CA, USA) located in the Translational Science Laboratory at the College of Medicine, Florida State University. The pooled data were demultiplexed into individual sample data, and adapter primer sequences were removed (Song et al., 2019).

Total gene counts data was analysed by NetworkAnalyst 3.0. Genes with counts less than 10, variance less than 10% and unannotated were filtered, and the remaining were normalized by Log2‐counts per million. Differential expressed gene were identified by DEseq2. Heatmap of globe differential expressed genes and gene enriched pathways were also visualized by the same online tool. The genes that were upregulated and downregulated between 2D versus 3D groups were further assessed for GO, KEGG pathway and phenotype pathway analysis by g:Profiler (version e104_eg51_p15_3922dba).

### 4.10 Proteomics analysis of hMSC EVs

The hMSC 2D and 3D EVs were isolated using ExtraPEG and then extracted for proteins. Based on protein quantification result, up to 100‐μg proteins were isolated on an S‐trap micro column (Protifi, Farmingdale, NY, USA, K02‐micro). The isolated proteins (triplicate for each group) were alkylated and digested on column based on manufacturer's instructions. All the eluted peptides were fractionated by Pierce high pH reverse phase peptide fractionation kit (ThermoFisher Scientific, 84868) into five fractions for each sample. Then all the samples were vacuumed dried and submitted to the FSU Translational Science Laboratory. The samples were analysed on the Q Exactive HF Orbitrap LC‐MS/MS System (ThermoFisher Scietific) as previously described (Hurwitz & Meckes, 2017; Hurwitz et al., 2018). Briefly, resulting raw files were searched with Proteome Discoverer 2.4 using SequestHT, Mascot and Amanda as search engines. Scaffold (version 5.0) was used to validate the protein and peptide identity. Peptide identity was accepted if Scaffold Local FDR algorithm demonstrated a probability greater that 99.0%. Likewise, protein identity was accepted if the probability level was greater than 99.0% and contained a minimum of two recognized peptides. GO annotation was carried out by g:Profiler.

### 4.11 Mitochondrial mass (mito‐mass), mitochondria membrane potential (MMP), and total ROS measurements

For mitochondrial mass and MMP measurement, trypsinized hMSCs (hMSCs:EVs = 1:1000 (cell number vs. EV number) were washed in warm Hank's Balanced Salt Solution (HBSS). Cell suspension was incubated with MitoTracker green FM or tetramethylrhodamine methyl ester (Molecular Probe, Eugene, OR, USA) at 37°C for mito‐mass and MMP, respectively. Cells were washed with HBSS, and analysed by flow cytometry (BD Biosciences, San Jose, CA, USA). For ROS measurement, cell suspension was incubated with 25 μM carboxy‐H2DCFDA (Molecular Probe) at 37°C for 30 min, and total ROS was determined using flow cytometry.

### 4.12 Human fibroblast (hFB) growth and in vitro wound healing model

Primary human dermal fibroblasts (CS‐201‐012) were purchased from American Type Culture Collection (ATCC, Manassas, VA, USA) and subcultured in DMEM plus 10% FBS up to passage 15. In vitro wound healing assay was performed to evaluate the effects of hMSC EVs on cell proliferation and migration. Briefly, hFBs were seeded (0.2 × 10^6^ cell/well) onto tissue culture treated 24‐well plate and grown overnight. An artificial wound was introduced with a 200 μL pipette tip and. After creating the scratch and PBS washing, complete culture medium (α‐MEM and 1% Penicillin/Streptomycin) with 1% FBS were added for further culture. EVs then were added at the concentration 1 × 10^8^ EVs/ml medium for next 24 h. Images were captured with an Olympus IX70 inverted microscope for 0–24 h. Cell growth was then recorded and analysed by ImageJ to calculate the wound closure rate, which may be attributed to the combination of cell proliferation and migration.

### 4.13 Measurement of indoleamine 2,3‐dioxygenase (IDO) activity

For IDO enzymatic activity, both IDO1 and IDO2 (both convert Tryptophan to Kynurenine) were assessed by measuring Kynurenine level in cell culture supernatant. A 400 μL supernatant from hMSCs culture (either stimulated by IFN‐γ at 40 ng/ml or left unstimulated for 24 h until sample collection) was clarified by mixing with trichloroacetic acid (200 μL, 30% wt/vol; Sigma Aldrich, St. Louis, MO, USA) by vortex, followed by centrifugation at 8,000 g for 5 min. An equal volume of Ehrlich reagent (2% p‐dimethylaminobenzaldehyde in glacial acetic acid) was added to the clarified supernatant, and optical density at 490 nm was measured.

### 4.14 Macrophage polarization assay

THP‐1 cells were obtained from ATCC and cultured in ATCC‐formulated RPMI‐1640 until confluency. THP‐1 cells were passaged into 6‐well plates at a concentration of 1.0 × 10^6^ cells/well. THP‐1 cells were differentiated into macrophages (M0) by a 48‐h treatment of phorbol 12‐myristate 13‐acetate (PMA, 20 nM, Sigma). M0 macrophages were polarized into the M1 phenotype with another 24 h‐treatment of lipopolysaccharide (LPS) (100 ng/mL, Sigma) and IFN‐γ (50 ng/mL, PeproTech, Cranbury, NJ, USA), or M2 phenotype by IL4 (50 ng/ml, PeproTech). hMSC EVs were added to the culture at the concentration of 1 × 10^8^ EVs/ml medium at the beginning of M1 polarization or M0 cells. M1 or M2 polarization markers were tested by qRT‐PCR.

### 4.15 T cell proliferation assay

Human peripheral blood mononuclear cells (hPBMCs) were obtained from STEMCELL Technologies Inc. (Vancouver, Canada) which followed an IRB‐approved protocol. Cells are free of HIV, CMV and hepatitis B/C. hPBMCs were cultured in RPMI‐1640 supplemented with 10% FBS (Seradigm, Radnor, PA, USA; 1400–500), 2‐mM L‐glutamine (Corning; 25‐005‐Cl), 100 IU penicillin‐streptomycin (Corning; 30‐002‐CI) in a standard CO_2_ incubator (37°C and 5% CO_2_). After thaw and cultured for 24 h, cells were labeled with carboxyfluorescein succinimidyl ester (CFSE, BioLegend, San Diego, CA, USA, 423801) at 1.0 μM for 20 min at a cell concentration of 10^7^ cells/mL, protected from light and washed twice with culture medium. Then, hPBMCs were seeded at 1 × 10^6^ cells/mL and stimulated with anti‐CD3/CD28 antibodies (2.0 μg/ml, BioLegend, 317325/302933). After 24 h, 2D‐ and 3D‐hMSC‐EVs (5 μg and 15 μg total protein or 0.6, 1.8, 3.0 and 7.5 × 10^10^ EVs/mL) were added in triplicates and incubated for another 3 days. hPBMCs were spun down and stained with 7‐AAD and BV421 anti‐CD8 antibody (BioLegend, 344748) for cytotoxic T cell proliferation assay, as well as anti‐CD4 antibody (BioLegend, 317418) for helper T cell proliferation assay. Samples were run on FACSCanto flow cytometer (BD Bioscences) in the Translational Laboratory of the FSU College of Medicine. The data were analysed by FACSDiva software (BD Biosciences).

### 4.16 Statistical analysis

Unless otherwise noted, all experiments were performed at least three times in triplicate (*n* = 3), and the representative data are reported. Experimental results are expressed as means ± standard deviation (SD) of the samples. Statistical comparisons were performed by one‐way ANOVA and Tukey's post hoc test for multiple comparisons, and significance was accepted at *P* < 0.05.

## AUTHOR CONTRIBUTIONS

Xuegang Yuan: Conceptualization; Data curation; Investigation; Methodology; Visualization; Writing – original draft; Writing – review & editing. Li Sun: Data curation; Investigation; Methodology; Visualization; Writing – original draft; Writing – review & editing. Richard Jeske: Investigation; Methodology. Dingani Nkosi: Investigation; Methodology. Yuan Liu: Investigation; Methodology. Sara York: Investigation; Methodology. Samuel Grant: Funding acquisition; Project administration; Visualization; Writing – review & editing. Yan Li: Conceptualization; Funding acquisition; Methodology; Project administration; Resources; Visualization; Writing – original draft; Writing – review & editing. David Meckes: Conceptualization; Funding acquisition; Methodology; Project administration; Resources; Visualization; Writing – review & editing.

## CONFLICT OF INTEREST

Authors declare that they have no competing interests.

## Supporting information

SUPPORTING INFORMATION

## Data Availability

The datasets generated during and/or analysed during the current study are available from the corresponding authors on reasonable request. The RNA‐seq data have been deposited in NCBI's Gene Expression Omnibus and are accessible through GEO Series GSE185874. The mass spectrometry proteomics data have been deposited to the ProteomeXchange Consortium via the PRIDE partner repository with the dataset identifier PXD028975 an https://doi.org/10.6019/PXD028975.
