# Supplementary material for: Engineering extracellular vesicles by three‐dimensional dynamic culture of human mesenchymal stem cells
Source: J Extracell Vesicles. 2022 Jun 18;11(6):e12235. doi: 10.1002/jev2.12235 (PMC9206229; doi:10.1002/jev2.12235)
Supplement: Supplementary file 1 — SUPPORTING INFORMATION [file JEV2-11-e12235-s001.docx]

Supplementary Materials for

**Engineering Extracellular Vesicles by Three-dimensional Dynamic Culture of Human Mesenchymal Stem Cells**

Xuegang Yuan*, Li Sun, Richard Jeske, Dingani Nkosi, Sara B. York, Yuan Liu,

Samuel C. Grant, David G. Meckes, Jr*, Yan Li*

*Corresponding authors. Email: [yuanxg1989@g.ucla.edu](mailto:yuanxg1989@g.ucla.edu), [David.Meckes@med.fsu.edu](mailto:David.Meckes@med.fsu.edu), [yli4@fsu.edu](mailto:yli4@fsu.edu)

**This file includes:**

Supplementary Text

Figs. S1 to S8

Tables S1 to S6

**Other Supplementary Materials for this manuscript include the following:**

Data S1 to S7

Supplementary Text

*Osteogenic differentiation:* hMSCs were grown to confluence before complete culture medium (CCM) was switched to osteogenic differentiation medium containing high glucose DMEM (Gibco, Grand Island, NY), 10% fetal bovine serum (FBS), 1% penicillin/streptomycin, 100 mM dexamethasone, 10 mM sodium-β-glycerophosphate, and 0.05 mM ascorbic acid-2-phosphate. The media were changed every 2 days and the differentiation was maintained for 14 days. Cells were then collected for RT-PCR.

*Adipogenic differentiation:* hMSCs were grown to confluence before CCM was switched to adipogenic differentiation medium containing high glucose DMEM, 10% FBS, 1% penicillin/streptomycin, 0.2 mM indomethacin, 0.5 mM isobutyl-1-methyl xanthine, 1 µM dexamethasone, 10 µg/mL insulin, and 44 mM sodium bicarbonate. Medium was changed every 2 days and the differentiation was maintained for 14 days. Cells were then collected for RT-PCR.

Human adipose-derived stem cell (ASC) culture: Frozen ASCs at passage 1 were acquired from the Tulane Center for Stem Cell Research and Regenerative Medicine. The ASCs were isolated from the subcutaneous abdominal adipose tissue from three de-identified healthy donors that were younger than 45 years old with a body mass index lower than 25. The isolated cells were characterized for their MSC properties through colony-forming unit (CFU) assays as well as tri-lineage differentiation potential (osteogenic, adipogenic and chondrogenic differentiation) *in vitro*. Briefly, hASCs were seeded at a density of 1,500 cells/cm^2^ in 150 mm diameter Petri Dishes (Corning, Corning, NY) in a standard 5% CO_2_ incubator. Cells were cultured in CCM containing αMEM (Life Technologies, Carlsbad, CA) with 10% FBS (Atlanta Biologicals, Lawrenceville, GA), sodium bicarbonate (1X, ThermoFisher Scientific), and 1% Penicillin/Streptomycin (ThermoFisher Scientific) undergoing media changes every 2-3 days. Cells were grown to 80% conﬂuence and then harvested by incubation with 0.25% trypsin/EDTA (Invitrogen, Grand Island, NY) for 7 minutes. Harvested cells were sub-cultured on tissue culture surface or aggregated in ULA 6-well plates under wave motion for EV collection.

Umbilical cord (UC)-derived human MSC (UC-hMSC) culture: Frozen hMSCs derived from human umbilical cords are provided by SynergyBiologics (Tallahassee, FL). UC-hMSCs at passage 0 (P0) are cryopreserved and stored in liquid nitrogen. One vial of frozen UC-hMSCs (generally contains 1×10^6^ cells) was recovered by immediately thawing in a 37 ºC water bath. The cell suspension was diluted with CCM and centrifuged at 400 g for 5 min. After centrifugation, the cell pellet was resuspended with 1-3 mL CCM and plated onto 150-mm diameter petri-dish at 1200-1500 cells/cm^2^. The culture was maintained in a standard incubator (37 ºC, 5% CO_2_). Culture media were changed every two days with fresh CCM. For passaging, the UC-hMSCs are treated with 0.25% trypsin/EDTA solution at 37ºC for 5-7 min. Trypsin was neutralized by adding CCM and detached cells were collected in a 15 mL centrifuge tube. UC-hMSCs were pelleted at 500 g for 5 min and resuspended with CCM. The cell number was determined by hemacytometer. UC-hMSCs can be expanded on tissue culture surface or in ULA 6-well plates under wave motion for EV collection.

**
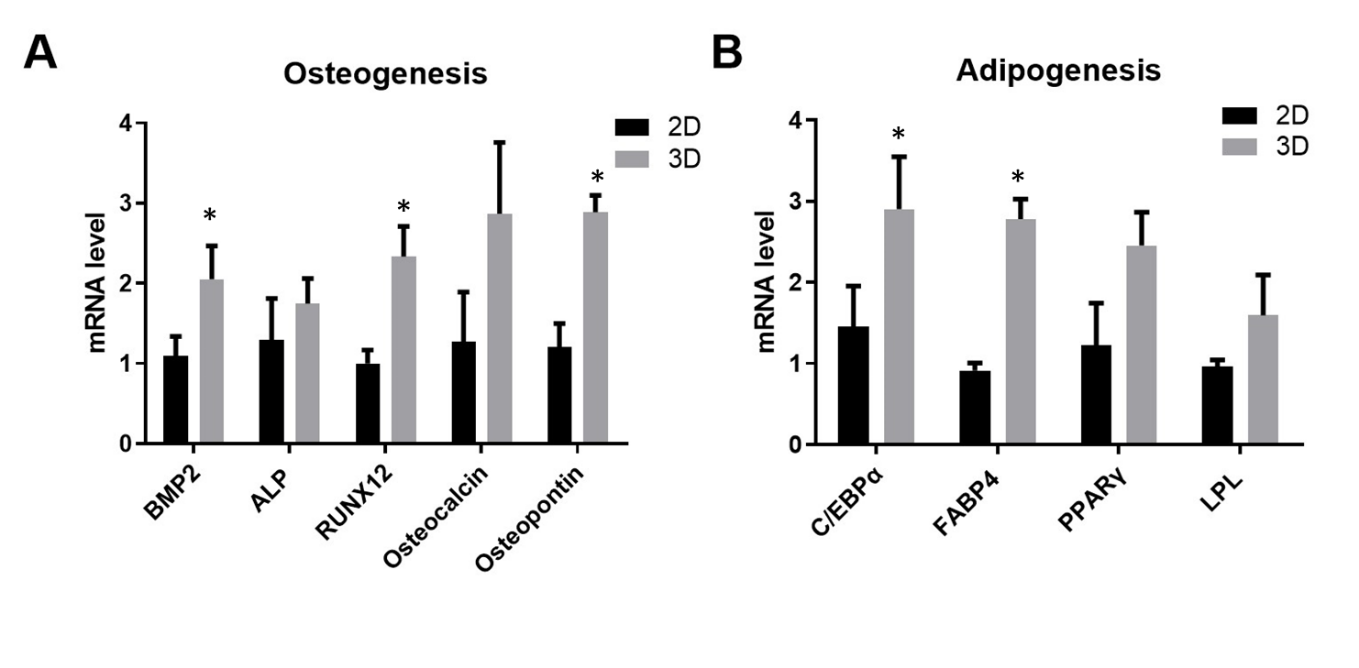
**

**Fig. S1. mRNA expression of lineage-specific differentiation markers of bone marrow-derived 3D hMSC aggregates.** mRNA expression was determined by RT-PCR. (A) Relative mRNA expression for osteogenic differentiation of 2D and 3D hMSCs; (B) Relative mRNA expression for adipogenic differentiation of 2D and 3D hMSCs. *, *p*<0.05 (n=3).

**
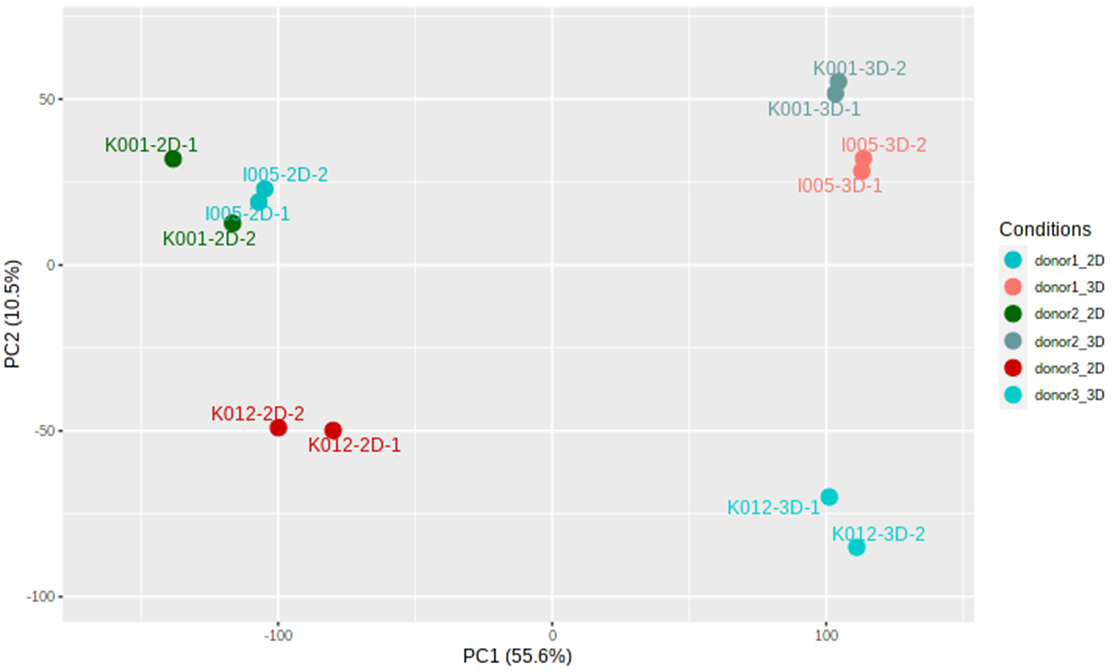
**

**Fig. S2. PCA plot of mRNA sequencing data from 12 libraries: different donors of UC-hMSCs in both 2D and 3D culture.**

**
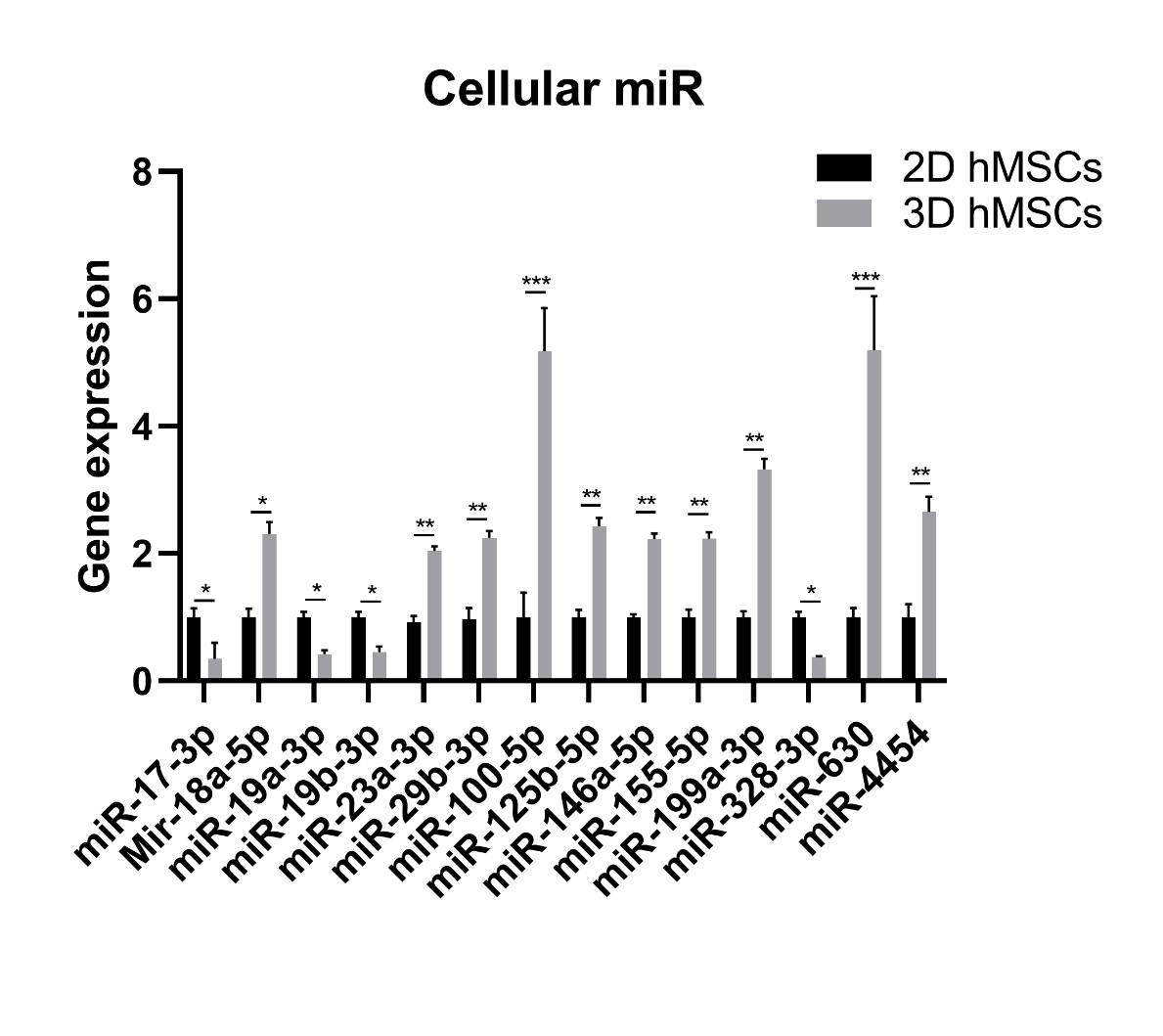
**

**Fig. S3.** **miRNA content determined by qRT-PCR in the cells was altered by 3D dynamic aggregate culture** (n=3). *, *p*<0.05; **, *p*<0.01; ***, *p*<0.001.


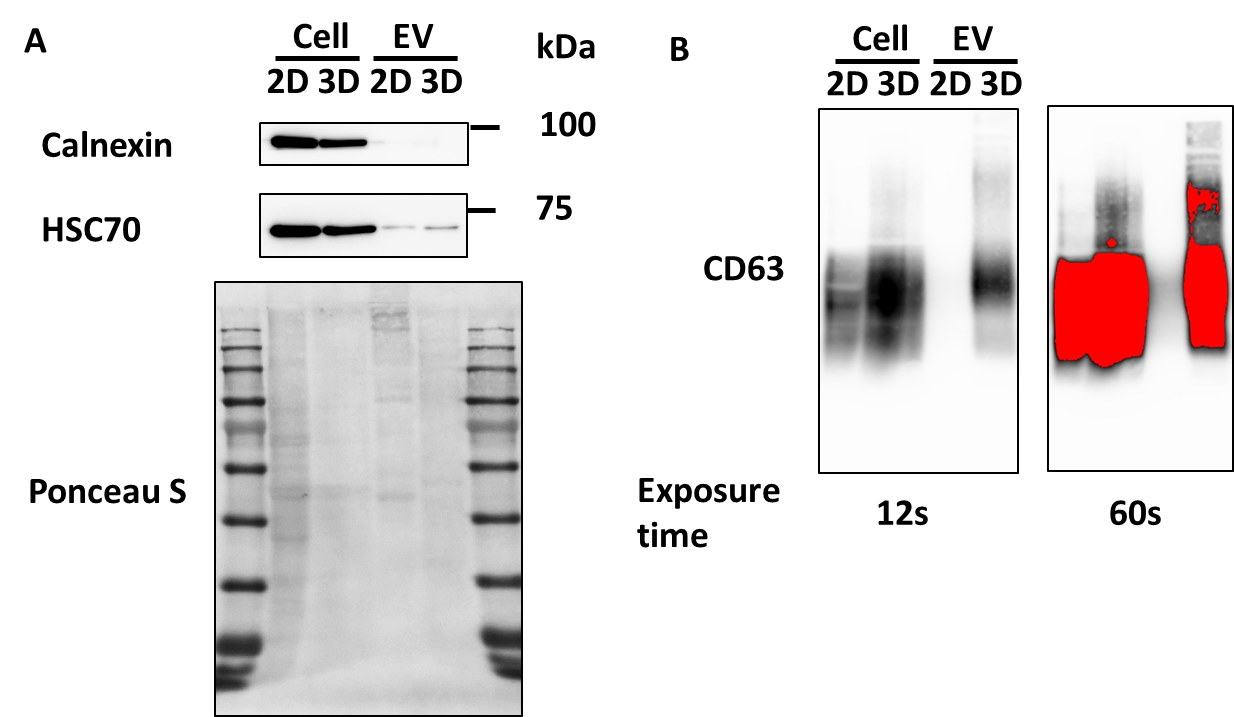


**Fig. S4. Additional results for CD63 and HSC70 expression in 2D- and 3D-hMSC-EV** **by Western Blot.** (A) Confirmation of negative Calnexin expression and weak HSC70 expression in hMSC-EVs; Ponceau S staining shows the equal protein loading. (B) Confirmation of weak CD63 expression for 2D-hMSC-EV. When the band for 2D-hMSC-EV is visible, the other bands would be overexposed.

**
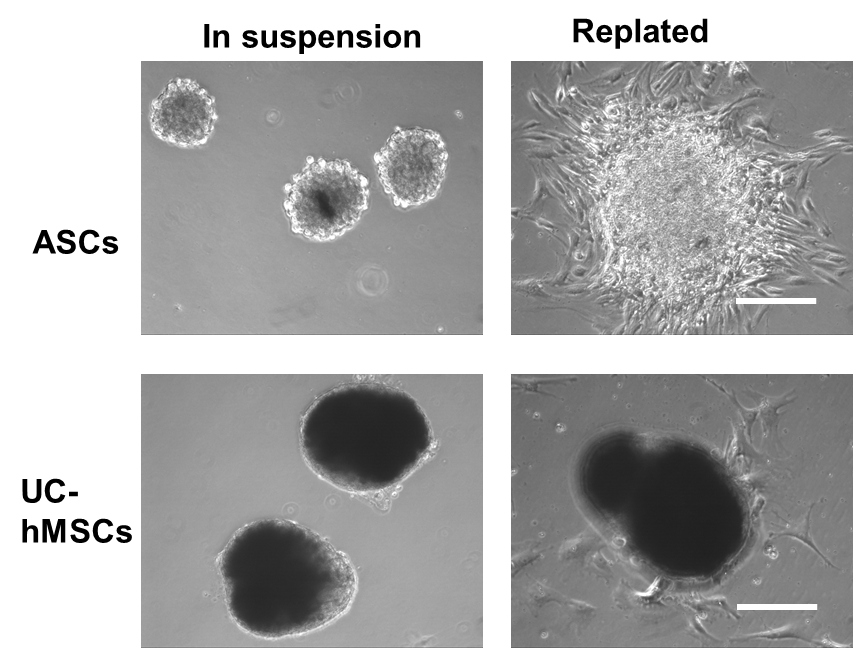
**

Fig. S5. Representative images of 3D aggregates for human ASCs and UC-hMSCs. Scale bar: 200 µm.


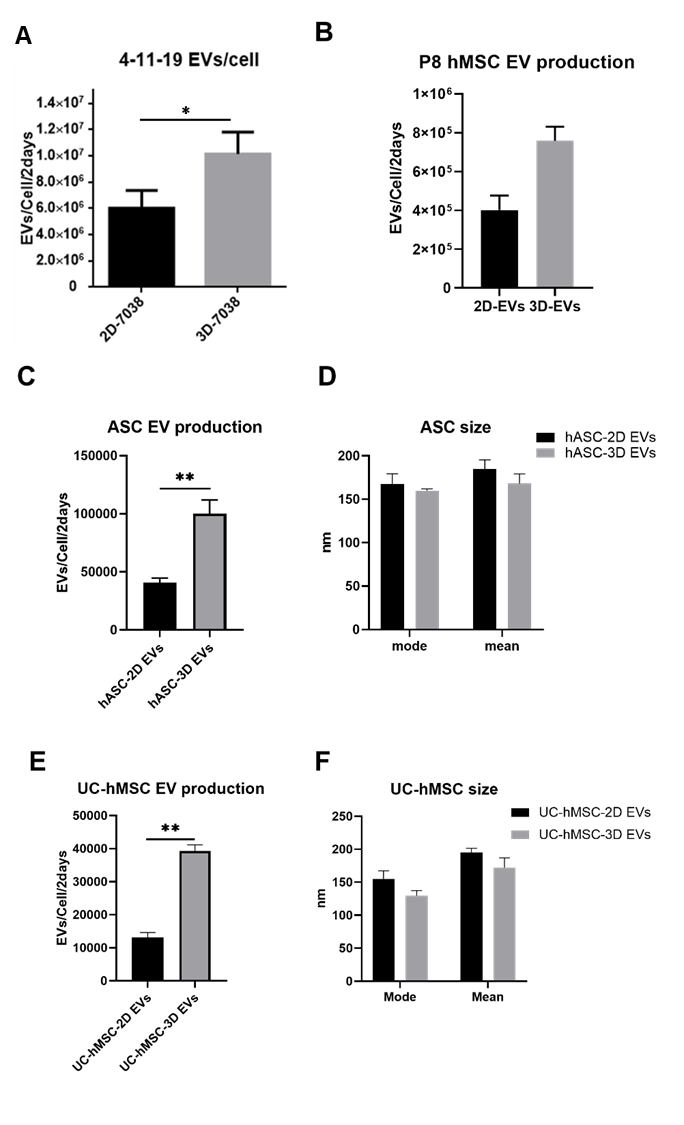


Fig. S6. 2D EV and 3D EV production from different hMSC donors, passages, and tissue sources. (A) EV production per cell for BM hMSCs of a different donor (n=3). (B) EV production per cell for BM hMSCs at passage 8 (n=3). (C) EV production from ASCs (n=3). (D) Mean and mode size of ASC-EVs determined by NTA (n=3). (E) EV production from UC-hMSCs (n=3). (F) Mean and mode size of UC-hMSC-EVs determined by NTA (n=3). *, p<0.05; **, p<0.01


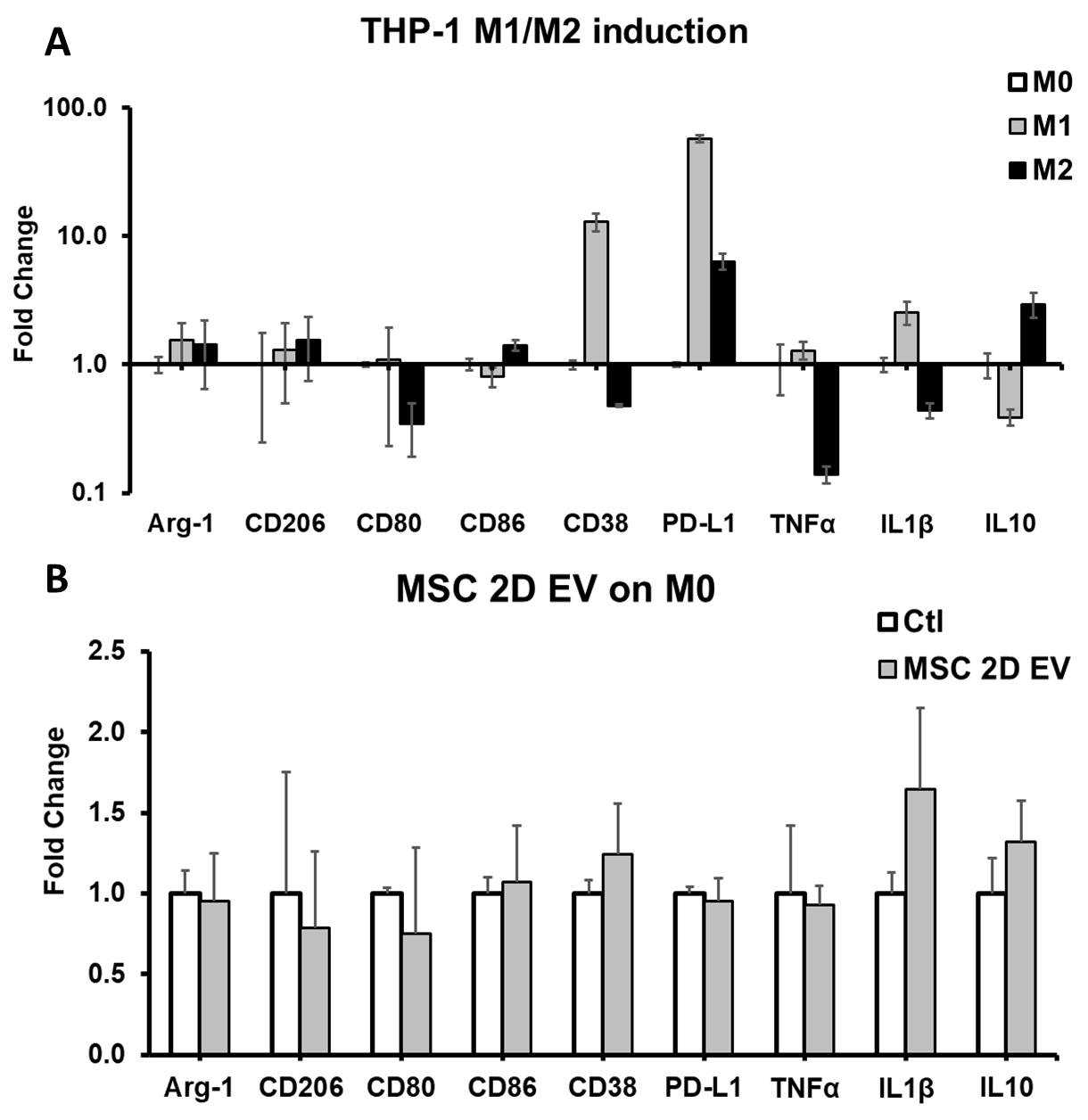


**Fig. S7. Additional RT-qPCR results for macrophage polarization assay.** Expression of M1/M2 phenotypic markers. **(A)** mRNA level of markers showing successful M1/M2 macrophage differentiation (n=3); **(B)** For M0 cells, hMSC-2D-EVs did not induce M1 or M2 polarization (n=3).

**
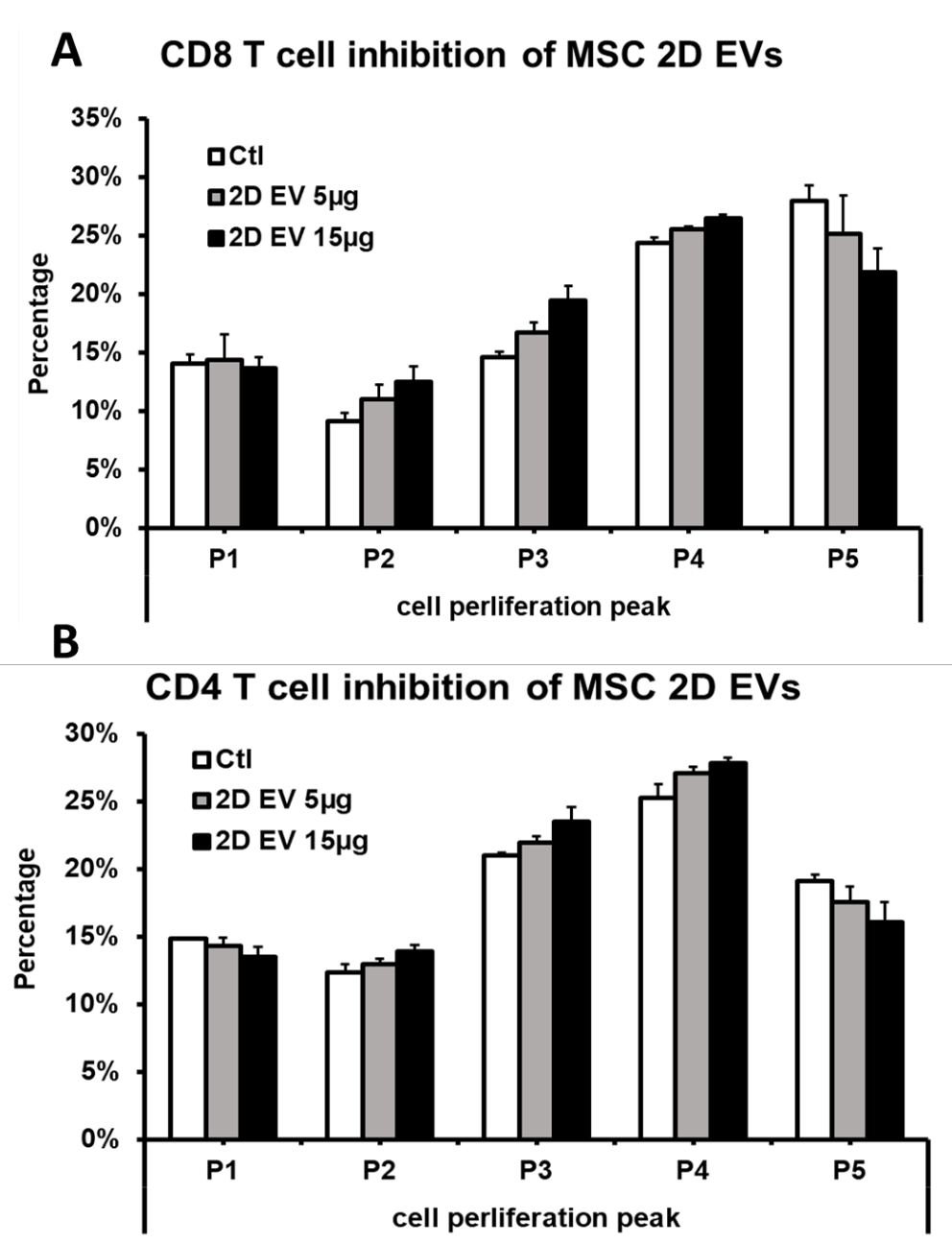
**

**Fig. S8. Additional results for T cell proliferation assay.** Peak percentages for (A) CD8+ T cells (n=3) and (B) CD4+ T cells (n=3). 2D-hMSC-EVs were evaluated at two doses, low dosage: 5 µg protein/group (or 0.58×10^10^ EV/mL); high dosage: 15.5 µg protein/group (or 1.8×10^10^ EV/mL).

| **Symbols** | **Name** | **logFC** | **P Value** |
| --- | --- | --- | --- |
| C2CD4A | C2 calcium dependent domain containing 4A | 8.17 | 3.4E-52 |
| C2CD4B | C2 calcium dependent domain containing 4B | 7.83 | 2.0E-60 |
| SMIM32 | small integral membrane protein 32 | 7.65 | 1.3E-32 |
| CXCR4 | C-X-C motif chemokine receptor 4 | 7.57 | 5.9E-37 |
| NTN1 | netrin 1 | 7.49 | 5.2E-55 |
| SLC16A6 | solute carrier family 16 member 6 | 7.42 | 8.6E-166 |
| BMP2 | bone morphogenetic protein 2 | 7.19 | 1.3E-112 |
| SPP1 | secreted phosphoprotein 1 | 7.00 | 4.9E-81 |
| GALNT9 | polypeptide N-acetylgalactosaminyltransferase 9 | 6.95 | 9.2E-56 |
| GPR84 | G protein-coupled receptor 84 | 6.89 | 7.1E-27 |
| APOD | apolipoprotein D | 6.82 | 8.1E-40 |
| MMP9 | matrix metallopeptidase 9 | 6.76 | 5.4E-19 |
| LINC02392 | long intergenic non-protein coding RNA 2392 | 6.74 | 9.6E-16 |
| MMP13 | matrix metallopeptidase 13 | 6.56 | 4.9E-35 |
| LINC01050 | long intergenic non-protein coding RNA 1050 | 6.44 | 1.0E-34 |
| TNFSF11 | TNF superfamily member 11 | 6.41 | 9.9E-77 |
| LGR6 | leucine rich repeat containing G protein-coupled receptor 6 | 6.31 | 1.2E-18 |
| TAC1 | tachykinin precursor 1 | 6.25 | 2.7E-16 |
| SFRP2 | secreted frizzled related protein 2 | 6.08 | 2.7E-33 |
| CXCL8 | C-X-C motif chemokine ligand 8 | 6.05 | 1.0E-23 |
| JPH2 | junctophilin 2 | -7.07 | 1.1E-29 |
| ANKRD1 | ankyrin repeat domain 1 | -6.47 | 6.9E-37 |
| LMOD1 | leiomodin 1 | -5.78 | 7.3E-48 |
| TENM2 | teneurin transmembrane protein 2 | -5.67 | 5.4E-52 |
| ACTC1 | actin alpha cardiac muscle 1 | -5.62 | 1.7E-19 |
| PLCE1-AS1 | PLCE1 antisense RNA 1 | -5.43 | 2.9E-12 |
| PTPRQ | protein tyrosine phosphatase receptor type Q | -5.43 | 5.6E-25 |
| MRVI1 | murine retrovirus integration site 1 homolog | -5.27 | 4.6E-43 |
| B3GALT2 | "beta-1,3-galactosyltransferase 2" | -5.24 | 1.5E-14 |
| LINC01018 | long intergenic non-protein coding RNA 1018 | -5.17 | 4.4E-21 |
| TENT5B | terminal nucleotidyltransferase 5B | -5.04 | 3.8E-15 |
| KRT19 | keratin 19 | -5.04 | 3.3E-51 |
| OXTR | oxytocin receptor | -5.04 | 5.7E-17 |
| LINC01085 | long intergenic non-protein coding RNA 1085 | -5.01 | 2.0E-19 |
| CNN1 | calponin 1 | -5.01 | 1.0E-44 |
| HRCT1 | histidine rich carboxyl terminus 1 | -5.00 | 4.2E-25 |
| SPAAR | small regulatory polypeptide of amino acid response | -4.98 | 2.6E-11 |
| DIAPH3 | diaphanous related formin 3 | -4.96 | 2.1E-35 |
| CCDC190 | coiled-coil domain containing 190 | -4.95 | 1.1E-15 |
| CPA4 | carboxypeptidase A4 | -4.89 | 3.1E-38 |

Table S1. The top 20 up- and down-regulated DEGs of hMSC RNA-seq.

| Symbols | Name | logFC | adj.P.Val |
| --- | --- | --- | --- |
| CXCL1 | C-X-C motif chemokine ligand 1 | 4.82 | 8.10E-13 |
| CXCL12 | C-X-C motif chemokine ligand 12 | -2.62 | 3.18E-13 |
| CXCL16 | C-X-C motif chemokine ligand 16 | 1.11 | 3.73E-02 |
| CXCL2 | C-X-C motif chemokine ligand 2 | 2.75 | 3.55E-06 |
| CXCL3 | C-X-C motif chemokine ligand 3 | 5.22 | 7.29E-14 |
| CXCL5 | C-X-C motif chemokine ligand 5 | 4.15 | 1.37E-06 |
| CXCL6 | C-X-C motif chemokine ligand 6 | 4.71 | 4.42E-16 |
| CXCL8 | C-X-C motif chemokine ligand 8 | 6.26 | 7.75E-25 |

**Table S2. The list of significantly upregulated CXCLs in 3D hMSC condition.**

| **Donor ID** | | **Age** | **BMI** | **Race** | **Sex** |
| --- | --- | --- | --- | --- | --- |
| **BM-hMSC** | 7038R | 30 | N.A. | Caucasian | F |
|  | 7051R | 33 | N.A. | Caucasian | M |
|  | 7052R | 20 | N.A. | Caucasian | F |
| **UC-hMSC** | I002 | 27 | 42.9 | N.A. | N.A. |
|  | I005 | 31 | 35.3 | N.A. | N.A. |
| **hASC** | ASC070522 | N.A. | N.A. | N.A. | N.A. |
|  | ASC506 | 43 | 24.8 | Caucasian | M |

N.A.: Not Available

Table S3. A summary of hMSC donor information.

| **Target** | **Host/ Isotype** | **Supplier/ Cat#** | **Dilution for Western** |
| --- | --- | --- | --- |
| Alix | Mouse monoclonal, IgG_1_ | Santa Cruz, sc-49268 | 1:1000 |
| HSC70 | Mouse monoclonal IgG_2a_ | Santa Cruz, sc-7298 | 1:1000 |
| Flotillin-2 | Mouse monoclonal, IgG_1_ | Santa Cruz, sc-28320 | 1:1000 |
| CD81 | Rabbit mAb (Mouse Specific) IgG | Cell Signaling, #10037 | 1:1000 |
| CD63 | Mouse monoclonal, IgG_1_ | Abcam; TS63 | 1:1000 |
| Syntenin-1 | Mouse monoclonal, IgG_1_ | Santa Cruz, S-31 | 1:1000 |
| CD9 | Mouse monoclonal, IgG_1_ | Millipore; MM2/57 | 1:1000 |
| Caveolin-1 | Mouse monoclonal, IgG_2b_ | Cell Signaling, #3267 | 1:1000 |
| Calnexin | Mouse monoclonal, IgG_1_ | Santa Cruz, S-70 | 1:1000 |

**Table S4.** **A list of antibodies used in Western Blot**

| **miR type** | **Primer Sequence** |
| --- | --- |
| miR-22-3p | CGAAGCTGCCAGTTGAAGAAC |
| miR-124-3p | ACGCGGTGAATGCCAAAA |
| miR-133b | GTTTGGTCCCCTTCAACCA |
| miR-181c-5p | AACATTCAACCTGTCGGTGAGT |
| miR-328-3p | CCCTCTCTGCCCTTCCGTA |
| miR-1246 | GCAATGGATTTTTGGAGCA |
| miR-21-5p | GCTAGCTTATCAGACTGATGTTGAAA |
| miR-17-3p | GCTAGCTTATCAGACTGATGTTGAAA |
| miR-18a-5p | AAGGTGCATCTAGTGCAGATAGAAA |
| miR-19a-3p | TGTGCAAATCTATGCAAAACTGA |
| miR-19b-3p | GTGCAAATCCATGCAAAACTG |
| miR-23a-3p | ACATTGCCAGGGATTTCCAA |
| miR-29b-3p | CGCTAGCACCATTTGAAATCAG |
| miR-100-5p | CCCGTAGATCCGAACTTGTG |
| miR-125b-5p | CTCCCTGAGACCCTAACTTGTG |
| miR-146a-5p | TGAGAACTGAATTCCATGGGTTA |
| miR-155-5p | TGCTAATCGTGATAGGGGTAAA |
| miR-199a-3p | CAGTAGTCTGCACATTGGTTAAAAA |
| miR-630 | GTATTCTGTACCAGGGAAGGTAAA |
| miR-4454 | CGAGTCACGGCACCAAA |
| SNORD48 | CTCTGAGTGTGTCGCTGATGC |
| SNORD44 | AACTGTGTGCTGATTGTCACG |
| Universal Forward primer | GCATAGACCTGAATGGCGGTA |

Table S5. Primer sequences for microRNAs in qRT-PCR analysis.

| **Gene** | **Forward primer 5' to 3'** | **Reverse primer 5' to 3'** |
| --- | --- | --- |
| Oct4 | CAGCAGATCAGCCACATCGCC | TGAGAAAGGAGACCCAGCAGCC |
| Nanog | CCTGTGATTTGTGGGCCTG | GACAGTCTCCGTGTGAGGCAT |
| Sox2 | GTATCAGGAGTTGTCAAGGCAGAG | TCCTAGTCTTAAAGAGGCAGCAAAC |
| CD9 | TCGCCATTGAAATAGCTGCGGC | CGCATAGTGGATGGCTTTCAGC |
| CD63 | ACAACCACACTGCTTCGATCC | GACTCGGTTCTTCGACATGGA |
| CD81 | ATCCTGTTTGCCTGTGAGGTG | TGCTGTAGGGCCTGGTCATAG |
| Alix (PDCD61P) | TAAGTGCATCTGAGGGCCAAA | GGGGCCTCCTTTCCTAGTTTC |
| ALIXI4 (PDCD61PI4) | TTGGCTAATCAGGCTGCAGAT | TCACATGCAAAGTAAGCAAGTGTT |
| TSG | CACCTGGTGGTCCATATCCTG | GATGGTGTCCTCGCTGATTGT |
| HRS | CTTCTCGGATCGAGGCTTCTT | TCGAATCAACTGCGCTCATTA |
| Syntenin | TTATTAAGCGGATGGCACCAA | TTGCCAAAGAAGGAAACTGGA |
| Rab27B | TCCATGAAGCTGCTTGTCTCA | GTTGGGTCTCCACCCAGAAAT |
| Rab27A | GCATGTTTCAGTTTTCAAGAACCA | AAAGGTGGCTTTTGTGTGTGC |
| 1SG15 | CTCTGAGCATCCTGGTGAGGAA | AAGGTCAGCCAGAACAGGTCGT |
| Flotillin1 | AAGCTGCCCCAGGTGGCAGAGG | TGTTCTCAAAGGCTTGTGATTCACC |
| KIBRA | CTCCGAGGCCAGAGCTGTAAGGAAC | CTAGAGGACTTGTGACTCAGTAC |
| MVP | TCCCTCTGGACCAAAATGAG | CCTTTTCCCACAGGACTTCA |
| Syndecan1 | TCGAATCTCTGTGCCTTCGTC | AAACCTTGGCTGAACCTACCG |
| TNFα | TGGCCAATGGCGTGGAGCTG | GTAGGAGACGGCGATGCGGC |
| IL-6 | GAACTCCTTCTCCACAAGCG | TTTTCTGCCAGTGCCTCTTT |
| IL-12β | CCAAGGGGTGACGTGCGGAG | GGTGGGTCAGGTTTGATGATGTCCC |
| IL-10 | AAGCCTGACCACGCTTTCTA | ATGAAGTGGTTGGGGAATGA |
| TGF-β | CCTACATTTGGAGCCTGGAC | TGTCCTTAAATACAGCCCCC |
| CD163 | CCAGTCCCAAACACTGTCCT | ATGCCAGTGAGCTTCCCGTTCAGC |
| CD206 | CTACAAGGGATCGGGTTTATGGA | TTGGCATTGCCTAGTAGCGTA |
| CD80 | GGGCACATACGAGTGTGTTGT | TCAGCTTTGACTGATAACGTCAC |
| CD86 | CTGCTCATCTATACACGGTTACC | GGAAACGTCGTACAGTTCTGTG |
| CD38 | TTCCCGCAGTTTTTCTTTGAA | GCACCTCTCAGCTGCCTACTG |
| Arg-1 | ACAGTTTGGCAATTGGAAGCA | CACCCAGATGACTCCAAGATCAG |
| PD-L1 | CCTACTGGCATTTGCTGAACGCAT | ACCATAGCTGATCATGCAGCGGTA |
| IL-1β | CCACAGACCTTCCAGGAGAATG | GTGCAGTTCAGTGATCGTACAGG |
| ALP | CAGTCTGCTGTGCCCCTGC | GTAGTTCTGCTCGTGGACGCC |
| BMP2 | TTCCCCGTGACCAGACTTTTGG | GCCACTTCCACCACGAATCCAT |
| Osteocalcin | GGCAGCGAGGTAGTGAAGAGAC | GAAAGCCGATGTGGTCAGCCAA |
| Osteopontin | AGCGGAAAGCCAATGATGAGAGC | ACTTTTGGGGTCTACAACCAGCAT |
| RUNX12 | CCAACCCACGAATGCACTATC | TAGTGAGTGGTGGCGGACATAC |
| CEBPA | AGCCTTGTTTGTACTGTATG | AAAATGGTGGTTTAGCAGAG |
| FABP4 | ACGAGAGGATGATAAACTGGTGG | GCGAACTTCAGTCCAGGTCAAC |
| LPL | CTGACCAAGGATAGTGGGATATAG | GGTAACTGAGCGAGACTGTGTCT |
| PPARγ | AGCCTGCGAAAGCCTTTTGGTG | GGCTTCACATTCAGCAAACCTGG |
| GAPDH | TCACTGCCACCCAGAAGACTG | GGATGACCTTGCCCACAGC |
| TUB1a  (TUBA1A) | TGAGGAGGTTGGTGTGGATTC | AAAAGCAGCACCTTTGTGACG |

Table S6. Primer sequences for target genes (mRNA) in qRT-PCR analysis.

Data file S1. Supplementary Excel Spreadsheet 1 for original mRNA sequencing data.

**Data file S2. Supplementary Excel Spreadsheet 2 for analyzed mRNA sequencing data.**

**Data file S3. Supplementary Excel Spreadsheet 3 for hMSC-EV proteomics Venn Diagram list.**

**Data file S4. Supplementary Excel Spreadsheet 4 for hMSC-EV proteomics sample support 2D and 3D groups.**

**Data file S5. Supplementary Excel Spreadsheet 5 for hMSC-EV proteomics g:Profiler DE intersections.**

**Data file S6. Supplementary Excel Spreadsheet 6 for hMSC-EV proteomics g:Profiler 3D intersections.**

**Data file S7. Supplementary Excel Spreadsheet 7 for Cell-RNA EV-protein functional analysis.**
